# Brusatol ameliorates psoriatic dyslipidemia by targeting IL-1β to restore AMPK-mediated lipid homeostasis

**DOI:** 10.1186/s13020-025-01287-8

**Published:** 2026-01-08

**Authors:** Yuankuan Jiang, Shumeng Zhang, Hewen Guan, Kejia lv, Jinchao Yu, Siyi Li, Renchuan Jia, Xiujie Zhang, Shurong Ma, Jialin Qu, Jingrong Lin

**Affiliations:** 1 Laboratory of Integrative Medicine, The First Affiliated Hospital of Dalian Medical University, No. 222, Zhongshan Road, Dalian, 116011 China; 2 Department of Dermatology, The First Affiliated Hospital of Dalian Medical University, No. 222, Zhongshan Road, Dalian, 116011 China

**Keywords:** Psoriasis, Dyslipidemia, Brusatol, IL-1β, AMPK signaling pathway, Lipid homeostasis

## Abstract

**Background:**

Psoriasis-associated dyslipidemia presents as a critical comorbidity characterized by a self-perpetuating cycle of metabolic dysfunction and chronic inflammation. Current treatment paradigms lack the capacity to simultaneously modulate these interconnected pathological axes. Here we investigated the efficacy and mechanisms of brusatol (a natural quassinoid derived from Bruceae Fructus) against psoriatic dyslipidemia from the insight of restoring lipid homeostasis.

**Methods:**

The in vitro efficacy of brusatol was assessed in TNF-α-stimulated HaCaT keratinocytes by evaluating proliferation, apoptosis, and inflammatory responses. In vivo, its therapeutic activity and systemic toxicity were examined in an imiquimod-induced psoriatic mouse model using PASI scoring, histopathological analysis, serum biochemical markers (TC, TG, ALT, AST, Cre), inflammatory cytokines (TNF-α, IL-1β, IL-17A), and BBB-chip analysis. Integrated proteomics and lipidomics of skin tissue and serum revealed dysregulated pathways, and subsequent target engagement was confirmed via molecular docking, CETSA, and DARTS. Mechanistic investigations included IL-1β overexpression, Co-IP, GST pull-down and AMPK pathway analysis (Western blot, qPCR) was explored to delineate the regulatory mechanisms.

**Results:**

Brusatol dose-dependently suppresses proliferation and inflammatory mediator expression in TNF-α-induced HaCaT keratinocytes, ameliorates skin lesions and systemic dyslipidemia in mice, effectively normalizing serum TC and TG levels without inducing visceral organ toxicity. Further integrated omic analyses and subsequent target validation identified IL-1β as the direct target linking inflammatory signaling and lipid dysregulation. Mechanistic studies uncovered a novel IL-1β–AMPK physical interaction that sequesters AMPK in the cytoplasm. Brusatol disrupts this complex, facilitating AMPK nuclear translocation to suppress lipogenic regulators (SREBP-1c/FASN/ACC1) and potentiate β-oxidation pathways (PPARα/CPT1A), thereby restoring lipid homeostasis.

**Conclusion:**

Our findings not only establish brusatol as an effective agent for ameliorating psoriatic dyslipidemia, but also unveil a fundamental IL-1β–AMPK interaction that orchestrates inflammation-metabolism crosstalk.

**Graphical Abstract:**

**Figure:**
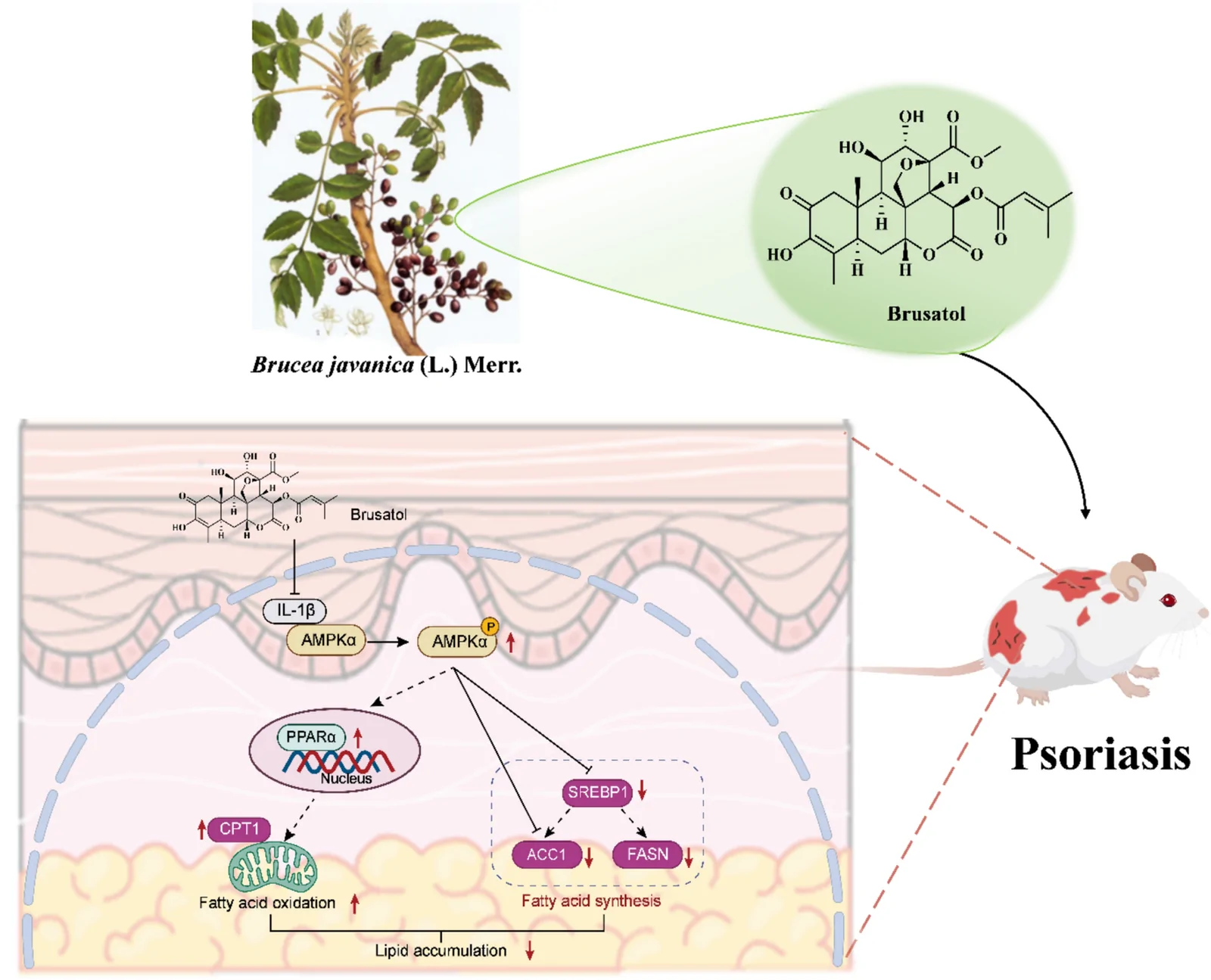

**Supplementary Information:**

The online version contains supplementary material available at https://doi.org/10.1186/s13020-025-01287-8.

## Highlights

- Brusatol dose-dependently suppresses proliferation and inflammation in TNF-α-induced HaCaT keratinocytes.
- Brusatol ameliorates skin lesions and systemic dyslipidemia in IMQ-induced mice models without inducing visceral organ toxicity.
- Brusatol ameliorates psoriatic dyslipidemia through direct binding to IL-1β, which disrupts a novel IL-1β–AMPK interaction and restores AMPK-mediated lipid homeostasis.

**Supplementary Information:**

The online version contains supplementary material available at https://doi.org/10.1186/s13020-025-01287-8.

## Introduction

Psoriasis is defined as a chronic, relapsing, inflammatory and systemic disorder mediated by the immune system and triggered by a combination of genetic and environmental factors [1]. It affects approximately 2–3% of the global population, demonstrating a steadily escalating prevalence trend annually [2]. Beyond its well-documented dermatological manifestations, psoriasis has increasingly been recognized as a systemic disorder associated with multiple comorbidities, including arthritis, diabetes, and lipid metabolism disorders. Among these, systemic dyslipidemia—marked by elevated serum triglycerides (TG), total cholesterol (TC), and low-density lipoprotein cholesterol (LDL-C)—exhibits the highest prevalence [3]. Importantly, this dysmetabolism is primarily observed as a systemic phenomenon in the circulation, with limited evidence for primary alterations in subcutaneous adipose tissue biology [1, 2, 4]. Consequently, addressing this systemic dysmetabolism has emerged as a critical therapeutic strategy. Genetic studies reveal that shared susceptibilities and overlapping pathogenic genes exist between psoriasis and lipid metabolism disturbances [4]. Besides, clinical trials and animal experiments consistently demonstrate dysregulated lipid metabolism in both serum and skin tissues of psoriasis patients and psoriasiform mouse models [5]. However, the causal relationship between psoriasis and lipid metabolism disorders remains unclear. On the one hand, elevated plasma triglyceride levels increase psoriasis risk. While conversely, psoriasis patients demonstrate a markedly increased prevalence of dyslipidemia relative to healthy controls [6]. Meanwhile, studies also suggest that statins can ameliorate skin lesions in psoriasis mouse models [7]. Collectively, these findings underscore the intimate link between lipid metabolism and psoriasis, highlighting lipid modulation as a critical therapeutic target.

In the pathogenesis of psoriasis, the inflammatory milieu triggered by cytokines represents the fundamental pathophysiological hallmark. Among these mediators, interleukin-1 beta (IL-1β) notably serves as a unique dual regulator, concurrently modulating both inflammation cascades and lipogenesis. On one hand, beyond the established role of effector T cells in IL-1β secretion to activate the Th17-mediated inflammatory cascade, keratinocytes can exacerbate inflammation in psoriatic lesions via autocrine IL-1β, thereby facilitating neutrophil infiltration [8]. On the other hand, IL-1β serves as a "lipid accumulation switch" specifically targeting adipocyte progenitor cells to stimulate their differentiation, activate fatty acid synthesis, facilitate foam cell formation, and contribute to pathological lead to lipid accumulation and metabolic dysfunction [9]. Consequently, exploring IL-1β-targeting therapeutics agents and their downstream regulatory pathways holds significant importance for developing advanced psoriasis management strategies.

The AMPK pathway, a pivotal focus in lipid metabolism dysregulation, orchestrating the suppression of fatty acid synthesis and concurrent stimulation of fatty acid β-oxidation to mitigate pathological lipid accumulation. Research demonstrates that pathological inactivation of AMPKα in psoriasis patients accelerates keratinocyte hyperproliferation, whereas pharmacological reactivation of AMPKα signaling effectively suppresses aberrant epidermal growth and ameliorates psoriatic pathology [10]. Moreover, substantial research identifies AMPKα as a central therapeutic target for diverse metabolic diseases. Its downstream signaling axes, SREBP-1c/FASN and PPARα/CPT1A, critically governing fatty acid biosynthesis and β-oxidation, respectively [11]. Therefore, activating AMPK to restore lipid homeostasis represents a potential therapeutic strategy for psoriasis. Intriguingly, IL-1β exerts functionally antagonistic effects against the AMPK signaling pathway, characterized by reciprocal suppression in metabolic and inflammatory regulation. Nevertheless, the role of the AMPK pathway in psoriasis remains understudied, and the regulatory mechanisms linking IL-1β and AMPK are yet to be elucidated.

**Fig. 1:**
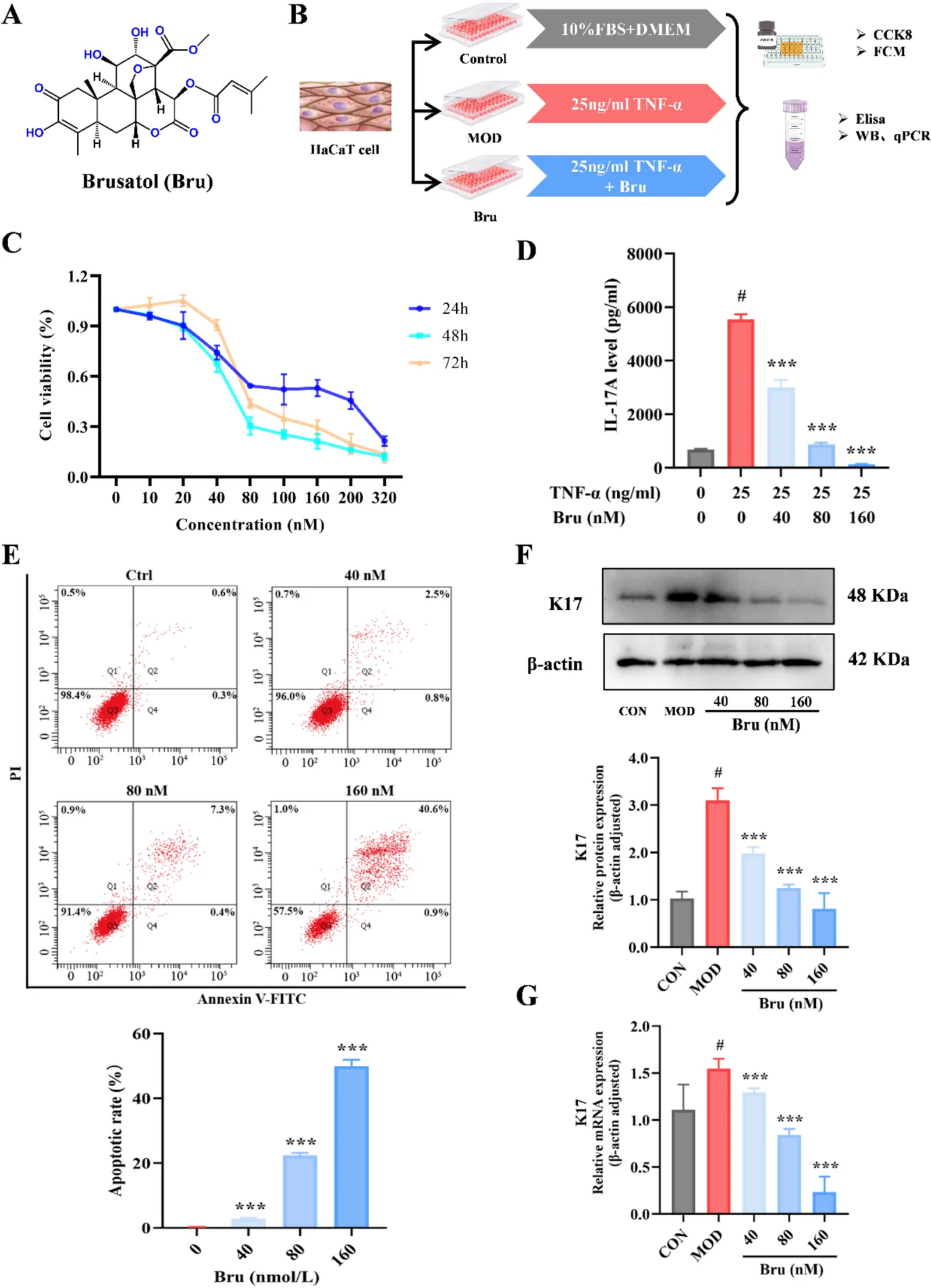
Brusatol inhibits proliferation, induces apoptosis, and reduces inflammatory marker expression in TNF-α-stimulated HaCaT keratinocytes. **A** Chemical structure of Brusatol. **B** Schematic representation of the in vitro cell-model induction protocol. **C** Cell viability was assessed by CCK-8 assay after treatment with Brusatol (0–320 nM) for 24, 48, and 72 h. Data are presented as percentage of vehicle control (0 nM). **D** Secreted IL-17A levels in cell culture supernatants were quantified by ELISA following 24 h treatment with Brusatol (40, 80, 160 nM) in TNF-α-stimulated HaCaT cells. **E** Representative flow cytometry plots (left) and quantitative analysis (right) of apoptosis assessed by Annexin V-FITC/PI double staining after 24 h treatment with Brusatol. The apoptotic rate represents the sum of early (Annexin V^+^/PI^−^) and late (Annexin V^+^/PI^−^) apoptotic cells. **F**, **G** Protein and mRNA expression levels of Keratin 17 (K17) following Brusatol treatment were analyzed by Western blot ( and RT-qPCR (**G**), with β-actin serving as the loading and reference control for both assays. Results (mean ± SEM, n = 3) are statistically compared to the TNF-α-stimulated model group (***p < 0.001); #*p<*0.01 versus the untreated control group.

In traditional Chinese medicine, psoriasis pathogenesis is attributed to blood deficiency with wind-dryness, driven primarily by exuberant heat-toxin accumulation. Consequently, therapeutic interventions principally employ bitter-cold herbs to purge pathogenic heat. Similarly, dyslipidemia manifests clinically as turbid dampness syndrome according to TCM pattern differentiation, for which bitter-flavored herbs constitute the core therapeutic agents. Brucea javanica is a representative cold-natured herb that is botanically from the dried ripen fruit of *Brucea javanica (L.)* Merr. Quassinoids, a class of tetracyclic triterpenoid bitter principles predominantly concentrated in this species, feature brusatol (chemical structure shown in Fig. 1A) as a primary constituent. Brusatol (C_26_H_32_O_11_, MW520.53) is a natural quassinoid compound classified under the Biopharmaceutics Classification System (BCS) as Class II, exhibiting the typical properties of low aqueous solubility (0.90 mg/mL) and high permeability (logP ≈ 2.4). Pharmacokinetically, it demonstrates limited oral bioavailability (8–15% in rodents) but achieves extensive tissue distribution, with particularly high concentrations observed in the lungs, spleen, and liver. Its clearance occurs primarily through CYP3A4/3A5-mediated hydroxylation and subsequent glucuronidation [12, 13]. Our previous investigations demonstrated compelling therapeutic efficacy of brusatol against malignant melanoma, primarily mediated through induction of apoptosis and suppression of the KLF4 signaling pathway [14]. Moreover, brusatol exhibits remarkable anti-inflammatory efficacy in murine models with ulcerative colitis, significantly diminishing serum levels of pro-inflammatory cytokines (e.g., IL-17 and TNF-α), inhibiting IL-22/STAT3 pathway activation, and facilitating intestinal mucosal repair [15]. Psoriasis pathogenesis is also primarily driven by dysregulation of IL-17 and TNF-α signaling pathways, with multiple biologics targeting these cytokines currently employed in clinical management. Consequently, modulation of these cutaneous inflammatory pathways supports the potential therapeutic efficacy of brusatol in psoriasis. Additionally, psoriasis is an inflammatory dermatosis mediated by Th17 cells, whose pathological differentiation is highly dependent on dysregulated fatty acid biosynthesis and aberrant cholesterol metabolism [16, 17]. Thus, it can be seen that brusatol holds promising therapeutic potential in regulating psoriasis with dyslipidemia. However, robust experimental evidence and the underlying mechanisms remain lacking.

Therefore, this study validates the therapeutic efficacy of brusatol for psoriasis using murine models and HaCaT keratinocyte cells, employing proteomics and lipidomics to identify IL-1β as a key target. Integration of molecular docking with drug affinity responsive target stability (DARTS) and cellular thermal shift assay (CETSA) confirms brusatol’s direct binding to IL-1β. Subsequent molecular biology experiments demonstrated that brusatol ameliorates psoriasis-associated dyslipidemia by modulating the AMPK-mediated SREBP-1c/FASN and PPARα/CPT1A signaling pathways. Collectively, these findings elucidate the pharmacodynamic mechanism of brusatol in treating psoriasis-linked lipid dysregulation, providing novel evidence for comorbidity regulation and offering valuable insights for expanding the clinical indications of brusatol.

## Materials and method

### Anti-proliferative, anti-inflammatory and safety assessment of brusatol in vitro

#### Cell culture

HaCaT cells (CL0090, Procell) were cultured in high-glucose DMEM medium (PM153210, Procell) supplemented with 10% fetal bovine serum (FBS-UE500, Newzerum) and 1% penicillin–streptomycin (P1400, Solarbio). The cells were incubated at 37 °C in a humidified 5% CO₂ atmosphere.

#### Cell viability and apoptosis assay

HaCaT cells were meticulously seeded into 96-well plates at a density of 4000 cells/well and allowed to adhere for 24 h. Subsequently, the cells were treated with brusatol (B26016, Shanghai Yuanye Bio-Technology Co., Ltd.) at graded concentrations (10–320 nM), with vehicle control (0 nM brusatol) included. After 24 h incubation, cell viability was detected using CCK-8 assay kit (MA0225, Dalian Meilun Biotech Co., Ltd) according to the manufacturer’s protocol.

As for the apoptosis assay, cell suspensions were harvested, washed twice with ice-cold PBS, and resuspended in 1 × Annexin V binding buffer. Subsequently, cells were co-stained with Annexin V-FITC and propidium iodide (PI) according to the manufacturer’s protocol (E-CK-A211, Elabscience^®^) followed by 15-min incubation at room temperature in darkness.

All samples were maintained on ice and analyzed within 60 min by flow cytometry (BD FACSCanto™ II). Apoptotic cells were quantified as the sum of Annexin V⁺/PI⁻ (early apoptosis) and Annexin V⁺/PI⁺(late apoptosis) populations.

#### Enzyme linked immunosorbent assay (ELISA)

The concentration of IL-17A in HaCaT cell supernatants was measured using an ELISA kit (Human, E-EL-H5812, Elabscience®) according to the manufacturer’s protocol.

### Safety assessment of brusatol in healthy mice

#### Animal model and study design

A cohort of twenty healthy male C57BL/6 mice (6–8 weeks, 22 ± 2 g) were randomly divided into two groups (n = 10 per group): a control group (CON) receiving saline vehicle and a treatment group (Bru) administered brusatol (9.2 mg/kg) via oral gavage for seven consecutive days. Body weight measurements were recorded daily. On day 8, blood samples were collected via the orbital plexus under anesthesia, after which the mice were euthanized for the collection of major organs (heart, liver, spleen, lungs, kidneys, and brain) for subsequent histopathological examination. All animal procedures were conducted in accordance with protocols approved by the Animal Ethics Committee of Dalian Medical University (Approval No. AEE19031).

#### Serum biochemical analysis

Serum levels of alanine aminotransferase (ALT, C009-2–1), aspartate aminotransferase (AST, C010-2–1), and creatinine (CRE, C011-2–1) were measured using commercial kits from Nanjing Jiancheng Bioengineering Institute following the manufacturer’s instructions.

#### Histopathological examination

The harvested organ samples were immediately fixed in 4% paraformaldehyde solution for 48 h. Following routine dehydration and embedding in paraffin, tissues were sectioned into 4 μm slices. After deparaffinization and rehydration, the sections were stained with hematoxylin and eosin (H&E) for microscopic evaluation of any pathological changes.

#### Assessment of blood–brain barrier integrity in vitro

Brain microvascular endothelial cells (BMECs) and astrocytes were cultured to confluence in DMEM supplemented with 10% fetal bovine serum (FBS-UE500, Newzerum) and 1% penicillin–streptomycin. Transwell inserts (3 μm pore size, 24-well format, PET membrane) were coated with rat tail collagen I (50 μg/mL) at 37 °C for 1 h. For inverted BBB-chip reconstruction, 5 × 10^4^ BMECs (were seeded onto the abluminal (bottom) surface of the insert, while 1 × 10^5^ astrocytes were seeded onto the luminal (top) surface. The inserts were subsequently inverted and transferred into a custom perfusion chip with continuous flow (0.2 mL/min). Dynamic co-culture was maintained for 4 days at 37 °C under 5% CO₂. On day 4, 80 nM brusatol was administered to the vascular (bottom) compartment. Trans-endothelial electrical resistance (TEER) was measured using an EVOM2 chopstick electrode system, with values blank-subtracted and reported as Ω·cm^2^.

### Evaluation the efficacy and toxicity of brusatol in IMQ-induced psoriasis-like mice

#### Animal model and experimental design

The IMQ-induced psoriasis model was selected for its well-established ability to recapitulate key features of human psoriasis pathology, including epidermal hyperplasia, and a robust IL-23/Th17-dominant inflammatory response [18]. Brusatol dosing (4.6 and 9.2 mg/kg) was based on prior efficacy data [19] and validated in-house for an optimal therapeutic window.

Treatment was administered for 7 days post-model establishment, a standard duration for assessing therapeutic reversal.

Male BALB/C SPF-grade mice (6–8 week, 20 ± 2 g) were purchased from Liaining Changsheng Technology Co., Ltd. (Liaoning, China). The housing environment was maintained at a temperature of 22.5 ± 2 °C and a humidity of 50–70%. Mice were housed in separate cages with 6 mice per cage and had free access to water and food. After a 7-day acclimatization period, mice were randomly assigned to experimental groups (n = 10 per group) using a random number table. All procedures involved in this experiment were conducted in accordance with the requirements and standards of the Animal Ethics Committee of Dalian Medical University (License No. AEE19031).

Mice (n = 10/group) were randomized into six groups: control (CON), model (MOD), low-dose brusatol (BL, 4.6 mg/kg), high-dose brusatol (BH, 9.2 mg/kg), negative control (NC), and methotrexate (MTX, 1 mg/kg). All experimental groups (excluding controls) were subjected to daily topical administration of 62.5 mg 5% imiquimod cream (H20030129, Sichuan Mingxin Pharmaceutical Co., Ltd.) on shaved dorsal skin (2 × 3 cm) under isoflurane anesthesia during days 1–14. Following successful model establishment, CON and NC groups were administered 0.2 mL normal saline via daily oral gavage from days 15–21, BL, BH and MTX groups received an equivalent volume of brusatol or methotrexate (solution in saline) by the same route, whereas MOD mice received no further intervention. Skin lesions were monitored daily until study termination on day 21. At this endpoint, mice underwent euthanasia by cervical dislocation under deep anesthesia followed by collection of serum and lesional skin tissue for subsequent analyses.

#### Scoring severity of skin inflammation

The clinical Psoriasis Area and Severity Index (PASI) was applied to evaluate skin inflammation severity and psoriasis progression in mice. Following standard PASI criteria, three parameters including erythema, scaling and induration were assessed. Each parameter was scored on a 0–4 scale defined as follows. 0, absent; 1, mild; 2, moderate; 3, marked; 4, Severe.

#### Histopathological staining

Skin tissue and major visceral organs samples were procured from mice and promptly fixed in 4% paraformaldehyde solution. After fixation, the tissues were embedded in paraffin. Subsequently, the paraffin-embedded blocks were sectioned into slices with a thickness of 4 μm, deparaffinized, rehydrated through graded ethanol and stained with hematoxylin and eosin (H&E) following routine protocols.

#### Enzyme linked immunosorbent assay (ELISA) for TNF-α, IL-17A, IL-1β

ELISA kits supplied by Elabscience Biotechnology Co., Ltd. were utilized to determine the levels of TNF-α (Mouse, E-EL-M3063), IL-17A (Mouse, E-EL-M0047), IL-1β (Mouse, E-EL-M0037) in serum with commercial kits. All experimental procedures were performed in strict accordance with the instructions supplied with the kits.

#### Colorimetric determination of TC, TG, ALT, AST and CRE

Total cholesterol (TC, A111-1–1), triglycerides (TG, A110-1–1), alanine aminotransferase (ALT, C009-2–1), aspartate aminotransferase (AST, C010-2–1) and creatinine (CRE, C011-2–1) in serum were measured with commercial kits (Nanjing Jiancheng Bioengineering Institute) following the manufacturer’s brief instructions.

### *In vitro* hypolipidemic activity determination

#### Establishment an in vitro model of hepatic steatosis using oleic acid (OA)-induced HepG2 cells.

HepG2 cells (CL0103, Procell) were cultured in high-glucose DMEM (Gibco) supplemented with 10% FBS, 100 U/mL penicillin and 100 µg/mL streptomycin, maintained at 37 °C in a humidified 5% CO₂ atmosphere. To establish the steatosis model, cells were seeded in 24-well plates at a density of 4 × 10^5^ cells/mL and allowed to adhere for 24 h. Steatosis was induced by replacing the maintenance medium with fresh medium containing 100 µM oleic acid (dissolved in 0.5% fatty acid-free BSA), while control cells received vehicle alone. Following 24 h incubation, successful lipid accumulation was verified through Oil Red O staining and intracellular triglyceride measurement.

#### Oil Red O staining

After 24 h oleate treatment, cells were washed twice with ice-cold PBS, fixed with 4% paraformaldehyde (15 min, RT), and rinsed with distilled water. Sequential incubations were performed with 60% isopropanol (5 min) and freshly prepared Oil Red O solution (G1262, Solarbio; 10 min, 37 °C). Following four distilled water washes to remove unbound dye, nuclei were counterstained with Mayer’s hematoxylin (1 min) and cleared under running tap water.

### Decoding the mechanisms of brusatol in psoriasis treatment

#### Non-targeted proteomics

To characterize proteomic alterations in dorsal skin tissues of experimental mouse cohorts, we performed SWATH-MS-based label-free quantitative proteomic profiling. In brief, tissue specimens were homogenized, followed by total protein extraction via lysis at 4 °C for 30 min. Subsequently, 250 μg of protein was subjected to filter-aided sample preparation (FASP) protocol through 10-kDa centrifugal filters. Resultant peptides were fractionated and analyzed using an Eksigent nanoLC 400 coupled with TripleTOF 6600 system (AB Sciex, USA). Raw data were processed in MaxQuant (Matching UniProt database; FDR ≤ 0.01) to identify differentially expressed proteins using a significance threshold of *p* < 0.05.

#### Targeted lipidomics

Lipid metabolites were extracted by methyl tert-butyl ether/methanol/water (MTBE). Briefly, skin tissue homogenates were mixed with 120 μL methanol, followed by sequential addition of MTBE and ultrapure water with vortexing. After phase separation at 4 °C, the upper lipid phase (200 μL) was collected by centrifugation (12,000 × g, 10 min, 4 °C). The remaining fractions were pooled to prepare quality control (QC) samples and reconstituted in acetonitrile/isopropanol (1:1, v/v).

Lipid profiling was performed on an Ultimate 3000 UHPLC system coupled to a Q-Exactive mass spectrometer equipped with an Accucore C30 column (2.6 μm, 2.1 × 150 mm). Separation was achieved using a gradient elution with mobile phase A (60% acetonitrile in water) and B (10% acetonitrile in isopropanol containing 10 mM ammonium formate and 0.1% formic acid). MS analysis was conducted in both positive and negative ESI modes (m/z 300–2000) with a resolution of 70,000. Lipid identification was performed using LipidSearch 4.2 software against the LipidMaps database, followed by peak area extraction (TraceFinder 4.1) and statistical analysis after data preprocessing.

#### Molecular docking analysis

Four high-resolution (< 2 Å) crystal structures of human IL-1β (PDB entries: 5R8Q, 5R8E, 5R8M with bound ligands; 8RYS in apo form) were prepared by protonating at pH 7.4 (Protonate 3D), removing distal crystallographic water (> 5 Å from ligands), and repairing missing residues. Brusatol (PubChem CID 73432) underwent energy minimization employing the MMFF94x forcefield (RMS gradient ≤ 0.01 kcal·mol⁻^1^·Å⁻^1^). Docking utilized a genetic algorithm (300 population size, 30,000 generations) with GBVI/WSA ΔG scoring. For ligand-bound structures, the binding site was defined by co-crystallized ligand centroids (14 Å grid) for 8RYS, the top predicted pocket matching holo-structure sites was selected. Each protein–ligand pair underwent 100 independent runs, with resulting poses clustered at 2.0 Å RMSD. The top 10 poses by binding free energy (ΔG_bind) were retained for analysis.

#### Transient IL-1β overexpression in HaCaT keratinocytes

The pcDNA3.1( +)-FLAG-IL1B plasmid, which encodes human IL-1β (NM_000576.2), was purchased from YouBio Biotechnology Co., Ltd. (P0137). The inserted sequence was verified by Sanger sequencing and is available upon request. The empty pcDNA3.1( +) vector (V79020; Thermo Fisher Scientific, USA) was employed as the mock transfection control.

HaCaT cells were seeded in 6-well plates (5 × 10^5^ cells/well) 24 h prior to transfection (70–80% confluency). Cells were transfected with 2.5 µg of endotoxin-free pcDNA3.1( +)-FLAG-IL1B plasmid (NM_000576.2, sequence-verified) or empty vector (mock) using Lipofectamine 3000 in Opti-MEM, following manufacturer protocol with P3000 enhancer. After 6 h incubation, medium was replaced with fresh complete medium. Functional assays and protein harvests were performed 24–48 h post-transfection. Untransfected cells served as additional controls.

#### Co-immunoprecipitation (Co-IP) Assay and GST Pull-down Assay

Cells were lysed in ice-cold buffer for 30 min. After centrifugation (12,000 × g, 4 °C, 15 min), lysates were pre-cleared with Protein A/G-agarose beads (Pierce) at 4 °C for 1 h. After brief centrifugation, supernatants were incubated with 2 µg primary antibody or control IgG overnight at 4 °C, followed by 2 h incubation with fresh Protein A/G-agarose beads. Beads were washed extensively with lysis buffer and PBS, then boiled in 2 × SDS buffer for protein elution. Proteins were separated by 10% SDS-PAGE and transferred to PVDF membranes. After blocking with 5% non-fat milk/TBST, membranes were probed with primary antibodies (4 °C, overnight) and HRP-conjugated secondaries (RT, 1 h). Immunoreactive bands were detected by ECL and quantified using ImageJ.

To provide definitive evidence for the direct IL-1β-AMPK interaction suggested by prior co-immunoprecipitation studies, we conducted a GST pull-down assay with purified recombinant proteins. Briefly, purified GST-tagged AMPK and His-tagged IL-1β were co-incubated in a cell-free system. GST alone served as the negative control. Following incubation, the protein complexes were affinity-captured using glutathione-sepharose beads. After extensive washing to eliminate non-specific binding proteins, the pulled-down complexes were eluted and probed by immunoblotting with anti-His and anti-GST antibodies.

#### Reverse transcription-quantitative polymerase chain reaction (RT-qPCR)

Total RNA was extracted with AG RNAex Pro Reagent (AG21102, Accurate Biotechnology, China), and reverse transcribed using Evo M-MLV RT Kit (AG11705, Accurate Biotechnology, China) with gDNA removal. qPCR reactions employed SYBR Green Premix Pro Taq HS (AG11701, Accurate Biotechnology, China) on a BIOER 9600 system with gene-specific primers (Supplemental Table S1). All samples were quantitated using the 2^−ΔΔCT^ method by normalization to the level of β-actin.

#### Western blot (WB) analysis of HaCaT and skin tissues

Total protein extraction from cells and skin tissues was extracted using RIPA lysis buffer supplemented with both protease and phosphatase inhibitors, after which protein concentrations were determined by BCA assay according to the manufacturer’s protocol. Equal protein was resolved via 8–12% SDS-PAGE electrophoresis and transferred onto PVDF membranes. After blocking with 5% skim milk in TBST for 2 h at room temperature, the membranes were probed with primary antibodies overnight at 4 °C. Following three TBST washes, membranes were incubated with HRP-conjugated secondary antibodies (1:5000 dilution) for 2 h at room temperature. After additional TBST washes (3 × 10 min), protein bands were visualized using enhanced ECL (Tanon, 180–5001). Quantitative densitometric analysis was performed using ImageJ software with normalization to β-actin (Rabbit, AC038, ABclonal, 1:50000).

The primary antibodies including AMPKα (Rabbit, 66536–1-lg, Proteintech), p-AMPKα (Rabbit, 2535,CST), SREBP-1c (Rabbit, AF6288, Affinity), ACC1 (Rabbit, A19495, ABclonal), PPARα (Rabbit, 15540–1-AP, Proteintech), IL-1β (Rabbit, 16806–1-AP, Proteintech) and K17 (Rabbit, 13074, CST) were employed at a standardized dilution of 1:1000. While optimized dilutions of 1:5000 was selected for FASN (Rabbit,10624–2-AP, Proteintech) and CPT1A (Mouse, DF12213, Affinity).

#### Cellular thermal shift assay (CETSA)

HaCaT cell lysates (prepared in NP-40 buffer) were aliquoted and treated with 20 μM brusatol or DMSO (control) for 30 min at 4 °C. Samples were heated at graded temperatures (37–65 °C, 3 min) followed by 3-min cooling. Precipitated proteins were removed by centrifugation (20,000 × g, 20 min). Soluble IL-1β in supernatants was quantified by immunoblotting. Band intensities were analyzed with ImageJ and normalized to the 37 °C control.

#### Drug affinity responsive target stability (DARTS)

HaCaT lysates were incubated with brusatol (0, 0.25, 0.5, 1 μM) or DMSO for 1 h at 4 °C. Proteolysis was initiated by adding pronase (1:1000 w/w) for 30 min at 25 °C and terminated with 10 mM PMSF. Samples were resolved by SDS-PAGE, and IL-1β levels were detected by Western blot. Protein stability was quantified as band intensity relative to the no-pronase control.

### Statistical analysis

All statistical analyses were performed using GraphPad Prism software (version 9.5.0). Data are presented as mean ± standard deviation (SD) from a minimum of three independent replicates. For comparisons between two groups of normally distributed data, unpaired two-tailed Student’s t-tests were performed. When comparing more than two groups with equal variances, one-way ANOVA was employed. A *p*-value < 0.05 was considered statistically significant.

## Results

### Brusatol inhibits proliferation and inflammatory mediator expression in TNF-α-induced HaCaT cells

Psoriasis is a skin disease characterized primary by abnormal hyperproliferation and aberrant differentiation of keratinocytes, manifesting clinically as hyperkeratosis and parakeratosis, and accompanied by concomitant significant inflammatory cell infiltration. Accordingly, we established a psoriatic cell model using keratinocytes to evaluate the therapeutic effect of brusatol against psoriasis (Fig. 1B). CCK-8 assay results demonstrated that brusatol treatment dose-dependently inhibited the proliferative capacity of HaCaT cells, with a calculated IC_50_ value of 74.23 ± 14.17 nM (Fig. 1C). Consequently, the concentration of 80 nM was selected as the medium dose, with corresponding low (40 nM) and high (160 nM) doses was also applied for subsequent experimental interventions. To investigate the anti-inflammatory potential of brusatol, we quantified IL-17A levels in culture supernatants across treatment groups using ELISA. The results demonstrated that brusatol dose-dependently suppressed TNF-α-induced IL-17A production. After medium-dose brusatol treatment, IL-17A level in the supernatant decreased significantly from 5840.67 ± 223.61 ng/mL to 940.71 ± 86.23 ng/mL (*P* < 0.001) (Fig. 1D). Concomitantly, apoptosis assays also revealed that brusatol triggered apoptosis in a dose-dependent manner, with apoptotic rates progressively escalating at higher doses (Fig. 1E).

Notably, keratin 17 (K17) exhibits pathognomonic upregulation in psoriatic lesions, where both mRNA abundance and protein expression are markedly elevated compared to normal skin or eczema tissues. Consequently, we utilized K17 to evaluate the therapeutic efficacy of brusatol. Following modeling, K17 protein levels increased by 2.4-fold compared to normal controls, whereas brusatol intervention dose-dependently reduced K17 expression to 1.9-fold, 1.1-fold, and 0.9-fold of baseline levels, respectively. These reductions were paralleled by consistent transcriptional changes (Fig. 1F, G). These findings collectively demonstrate that brusatol inhibits keratinocyte apoptosis and inflammatory cytokine release.

### Brusatol demonstrates a favorable safety profile in healthy mice and effectively ameliorates both skin lesions and inflammation in IMQ-induced psoriatic mouse models

#### Safety assessment of brusatol in healthy mice

The systemic toxicity of brusatol was assessed by oral gavage of a high dose (9.2 mg/kg) to healthy C57BL/6 mice for seven consecutive days. Histopathological examination of major organs (heart, liver, spleen, skin, lungs, kidneys, and brain) revealed a complete absence of toxicological alterations such as necrosis, vacuolar degeneration, or inflammatory cell infiltration in brusatol-treated mice, with tissue morphology indistinguishable from that of the saline-control group (Fig.S1A).

Consistent with the histopathological findings, serum biochemical analysis further confirmed the lack of hepatotoxic or nephrotoxic effects, showing no significant alterations in ALT, AST, or CRE levels following brusatol treatment (Fig. S1B).

Given the reported neurotoxicity of other constituents from *Brucea javanica* (L.) Merr., we specifically investigated brusatol’s potential to disrupt the blood–brain barrier (BBB) using an in vitro organ-on-chip model. Treatment with 80 nM brusatol for 24 h did not compromise BBB integrity, as evidenced by the absence of notable changes in Trans-Endothelial Electrical Resistance (TEER) values compared to controls (Fig.S1C).

Collectively, these data establish that brusatol is well-tolerated at the tested dose and exhibits a favorable safety profile, with no discernible systemic or neurotoxic effects.

#### Therapeutic efficacy of brusatol in IMQ-induced psoriatic mice

**Fig. 2:**
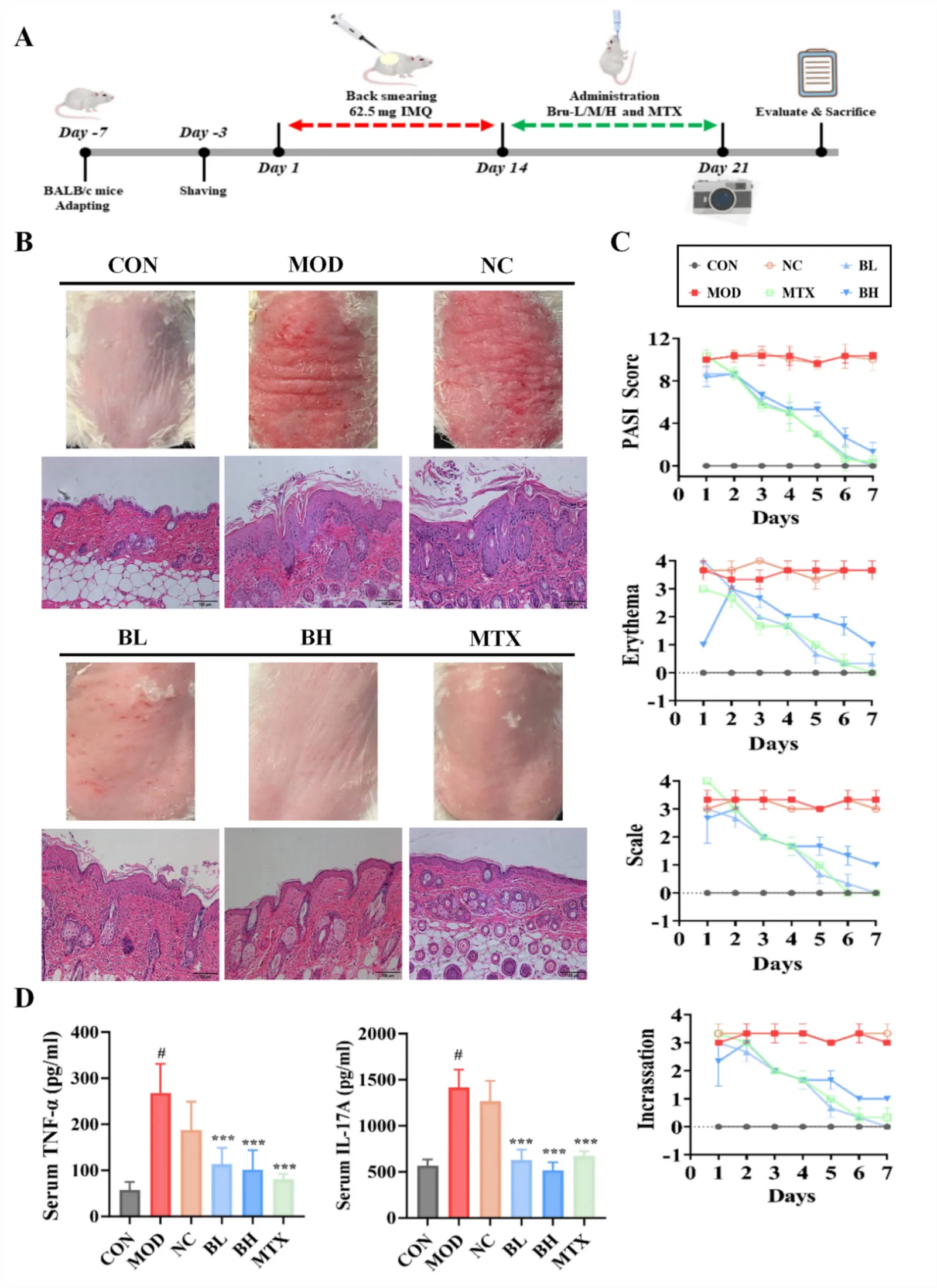
Brusatol treatment ameliorates skin lesions and reduces inflammatory cytokines in IMQ-induced psoriatic mouse model. **A** Schematic illustration of the in vivo experimental design. Mice received topical IMQ cream on shaved back skin for 14 days to induce psoriasis, followed by oral administration of brusatol (BL: 4.6 mg/kg; BH: 9.2 mg/kg), Methotrexate (MTX: 1 mg/kg), or vehicle for 7 days. **B** Representative clinical photographs (upper panel) and corresponding H&E-stained histological sections (lower panel) of dorsal skin from each group. Scale bar, 100 μm. **C** Dynamic changes in total PASI score (left) and subscores (right) throughout the 7-day treatment period. **D** Serum TNF-α and IL-17A levels measured by ELISA. Data are presented as mean ± SD (n = 10 mice/group). ***p < 0.001 versus the IMQ-induced model group; #*p*<0.01 versus the control group.

Following comprehensive safety assessment, we established an imiquimod (IMQ)-induced murine psoriasis model to systematically evaluate the in vivo therapeutic efficacy of brusatol (Fig. 2A). Observation of skin lesions and H&E staining results revealed that brusatol treatment significantly improved the epidermal condition. Specifically, treated mice exhibited epidermal thinning, reduced intercellular edema, resolution of hyperkeratosis, reappearance of the granular layer, and a substantial reduction in inflammatory cell infiltration within the dermis and around blood vessels (Fig. 2B). Daily Psoriasis Area Severity Index (PASI) scoring throughout the 7-day intervention period revealed brusatol’s comprehensive anti-psoriatic efficacy across all three PASI dimensions, evidenced by marked reductions in erythema area, scaling extent, and skin thickness (Fig. 2C).

Furthermore, serum concentrations of proinflammatory cytokines TNF-α and IL-17A were measured using ELISA to evaluate the anti-inflammatory effects of brusatol. As exhibited in Fig. 2D, the blank control group exhibited baseline levels of 57.11 ± 17.66 ng/mL (TNF-α) and 568.18 ± 66.58 ng/mL (IL-17A), respectively. In IMQ-induced psoriatic models, these cytokines were markedly elevated to 267.86 ± 65.35 ng/mL (TNF-α) and 1417.29 ± 191.45 ng/mL (IL-17A), confirming inflammatory pathogenesis (*p* < 0.001 vs. control). Treatment with low-dose brusatol reduced serum TNF-α and IL-17A levels to 113.31 ± 35.59 ng/ml and 623.93 ± 111.67 ng/ml, respectively (*p* < 0.001 vs. model). High-dose treatment induced a substantially greater suppression, achieving 61.8% and 63.4% reductions in TNF-α and IL-17A respectively compared to the model group (*p* < 0.001), whose therapeutic efficacy is comparable to methotrexate (positive control) in all evaluated parameters.

Notably, the low-dose regimen demonstrated efficacy comparable to higher doses, prompting its selection for subsequent mechanistic investigations.

### Brusatol significantly reverses the protein profile in IMQ-induced psoriasiform skin lesions

**Fig. 3:**
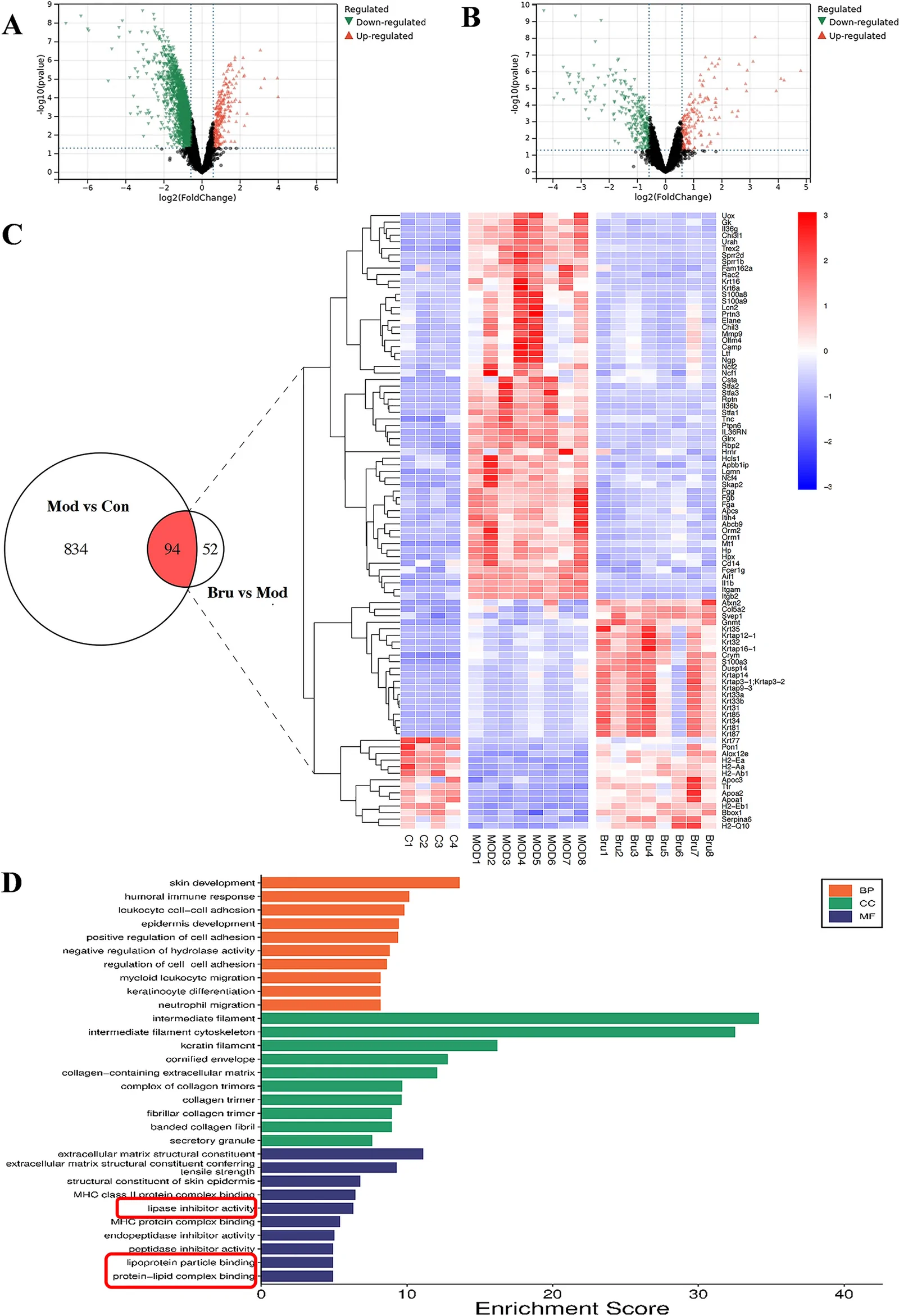
Brusatol reverses the IMQ-induced dysregulation of the cutaneous proteome. **A**, **B** Volcano plots depicting differentially expressed proteins (DEPs) in dorsal skin tissues from the **A** IMQ-induced model (Mod) *vs.* control (Con) groups, and **B** Brusatol-treated (Bru) *vs.* Mod groups. DEPs were defined by |log₂(fold change)|≥ 1 and FDR-adjusted *p* < 0.05 (red: up-regulated; blue: down-regulated). **C** Venn diagram illustrating the overlap of DEPs between the two comparisons. The heatmap displays the z-score normalized expression levels of the 94 DEPs commonly dysregulated by IMQ and reversed by Brusatol. Key proteins implicated in inflammation (IL-1β, CXCL1, S100A8/A9), hyperproliferation (KRT6A), and barrier function (FLG, LOR, IVL) are highlighted. **D** Significantly enriched Gene Ontology (GO) biological process terms for the 94 reversed DEPs (hypergeometric test with FDR correction). Data are from n = 4 or 8 mice per group

To elucidate the regulatory mechanism of brusatol in IMQ-induced psoriasiform skin lesions, we conducted untargeted quantitative proteomic profiling by data-independent acquisition (DIA) to identify differentially expressed proteins (DEPs). A total of 4258 high-confidence proteins (FDR < 1%, unique peptides ≥ 2) were identified across three experimental groups. Comparative analysis between Mod and Con group revealed 980 DEPs meeting stringent thresholds (|log2FC|≥ 1, FDR-adjusted *p* < 0.05), including 834 upregulated and 94 downregulated proteins (Fig. 3A, B). Following brusatol intervention, the total number of DEPs was drastically reduced to 146 (Bru vs Mod). Among these, 52 proteins were restored to baseline expression levels comparable to untreated controls, while 94 proteins exhibited partial reversal—remaining dysregulated relative to Con but with reversed directionality (i.e., upregulated proteins became downregulated or vice versa).

The heatmap display of screened 94 DEPs revealed that key pro-inflammatory mediators (e.g., IL-1β, CXCL1) and keratinocyte hyperproliferation-related proteins (e.g., S100A8, S100A9, KRT6A) significantly enriched in the Mod group were markedly reduced following brusatol treatment (Fig. 3C). Specifically, their log₂ FC values decreased from an elevated range of 2.1–4.7 (Mod) to 0.2–0.8 (Bru group). Conversely, barrier-repair proteins downregulated in the Mod group (FLG, LOR, IVL) were restored to upregulated status following brusatol intervention, with log_2_FC values increasing from − 2.3 to − 0.4 (Bru group). Subsequent KEGG and GO enrichment analyses of these DEPs indicated significant enrichment in the IL-17 mediated signaling pathway, high-density lipoprotein binding, and epidermal cell (keratinocyte) differentiation processes (Fig. 3D). Collectively, these results demonstrate that brusatol effectively rectifies the dysregulated protein expression profile induced by IMQ in psoriasiform skin lesions, suggesting indicative of a pleiotropic therapeutic mechanism targeting both inflammatory cascades and epidermal barrier reconstitution.

### Lipidomics analysis reveal the regulatory mechanisms of brusatol in psoriasis

**Fig. 4:**
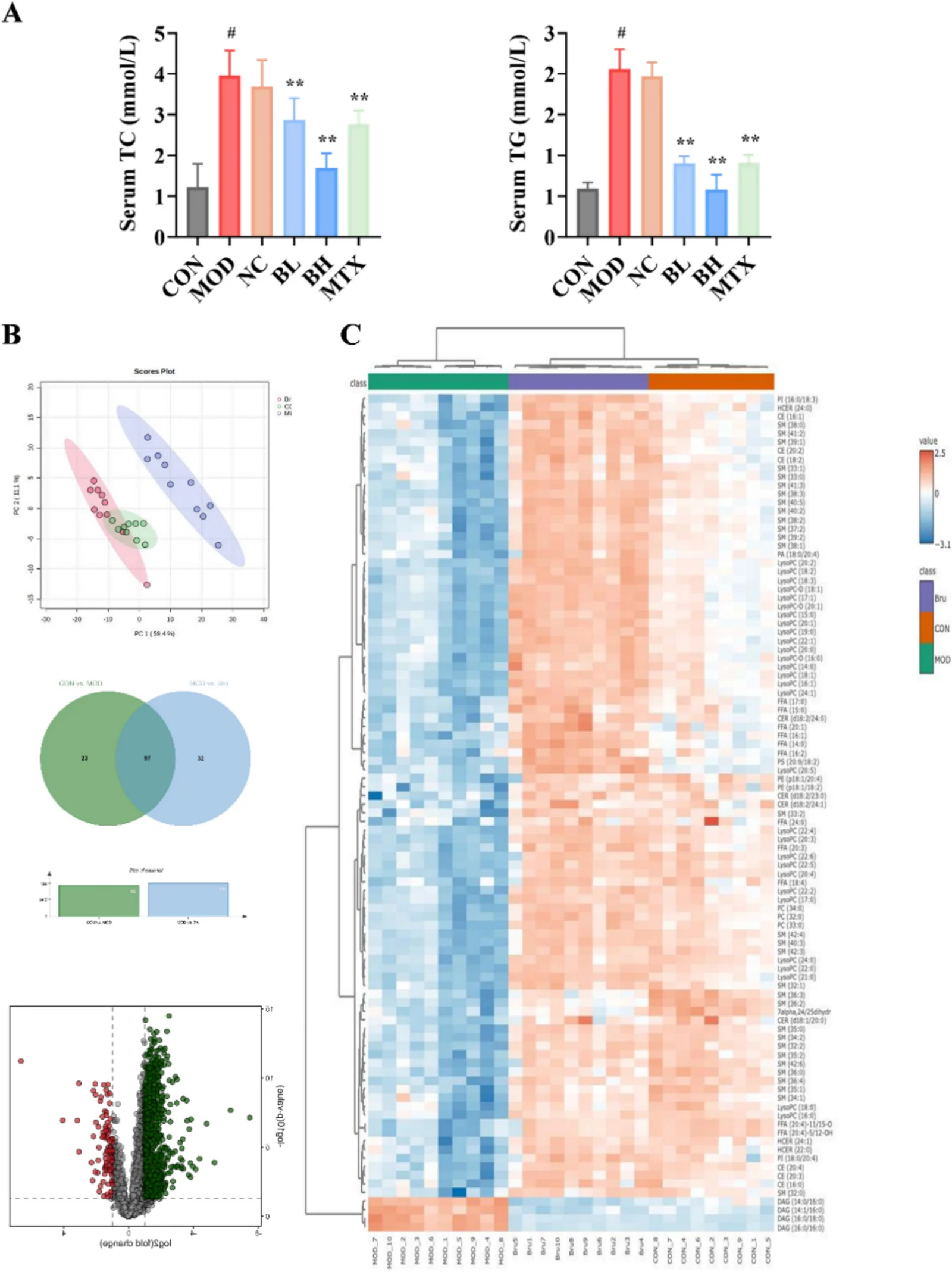
Brusatol restores systemic lipid homeostasis in IMQ-induced psoriasis mice. **A** Serum levels of total cholesterol (TC) and triglycerides (TG) across control (Con), model (Mod), Brusatol (BL: 4.6 mg/kg; BH: 9.2 mg/kg), and methotrexate (MTX: 1 mg/kg) treatment groups. Data are presented as mean ± SD (n = 10). *p < 0.05, **p < 0.01 versus the Mod group. **B** Lipidomic profiling. Left: PCA score plot showing group separation (Con, Mod, Bru). Middle: Venn diagram of differentially abundant lipids (fold change > 2.0 or < 0.5, VIP > 1.0, *p* < 0.05). Right: Volcano plot (Bru vs. Mod). **C** Hierarchical clustering heatmap of 97 significantly altered lipids. Lipid classes are annotated: *SM* sphingomyelin, *DAG* diacylglycerol, *LPC* lysophosphatidylcholine, *PC* phosphatidylcholine. **p < 0.01 vs. Mod group.  #*p*<0.01 vs. control group. Lipidomics data (B, C) are from n = 9 or 10 (n = 9 for CON group, n = 10 for all other groups) biologically independent samples per group

Building upon our proteomic screening results that implicated brusatol participated into lipid metabolism regulation, we detected serum total cholesterol (TC) and triglyceride (TG) levels across treatment groups to validate these findings (Fig. 4A). Notably, quantitative analysis revealed significant hyperlipidemia in the model group, with serum TC and TG levels elevated by 3.24-fold (3.96 ± 0.61 vs. 1.22 ± 0.57 mmol/L, *p* < 0.01) and 3.49-fold (2.06 ± 0.24 vs. 0.59 ± 0.08 mmol/L, *p* < 0.01), respectively, compared to healthy controls. Brusatol treatment dose-dependently reduced these atherogenic lipids (TC: up to 27.5% decrease; TG: up to 56.3% decrease, both *p* < 0.01 versus model), which correlated strongly with improvements in psoriatic lesion severity.

To elucidate the molecular mechanisms underlying brusatol’s regulation of lipid metabolism, we performed comprehensive lipidomic profiling of serum samples from controls, model and brusatol-treated group. Principal component analysis (PCA) revealed distinct clustering among the three groups (R_2_X = 0.82, Q2 = 0.75), with clear inter-group separation along PC1 (59.4% variance) and PC2 (11.1% variance) (Fig. 4B), indicating significant alterations in global lipid metabolism. Applying stringent criteria (VIP > 1.0, *p* < 0.05, and fold-change > 2.0 or < 0.5), 97 differentially expressed lipid species across all three groups were identified, with sphingomyelins (SM), diacylglycerols (DAG), lysophosphatidylcholines (LPC), cholesterol, and phosphatidylcholines (PC) enriched predominantly (Fig. 4C). Comparative analysis revealed a selective elevation of diacylglycerols (DAGs) in model group (2.4 ~ 5.6-fold increase, *p* < 0.01), while other measured lipid classes showed significant reductions relative to controls. SMs, LPCs and PCs are essential structural components of cellular membranes and play critical roles in cytoskeletal organization. Their significant depletion in the serum of psoriasiform mice likely reflects their increased utilization during pathological keratinocyte hyperproliferation, a hallmark of psoriatic pathogenesis. Contrary to expectations, we observed significant elevations in both DAG and cholesterol. These paradoxical findings prompted systematic evaluation of brusatol’s modulatory effects on glycerolipid metabolism and cholesterol homeostasis.

### Brusatol inhibits lipid synthesis and reduces lipid accumulation in steatotic HepG2 hepatocyte model

**Fig. 5:**
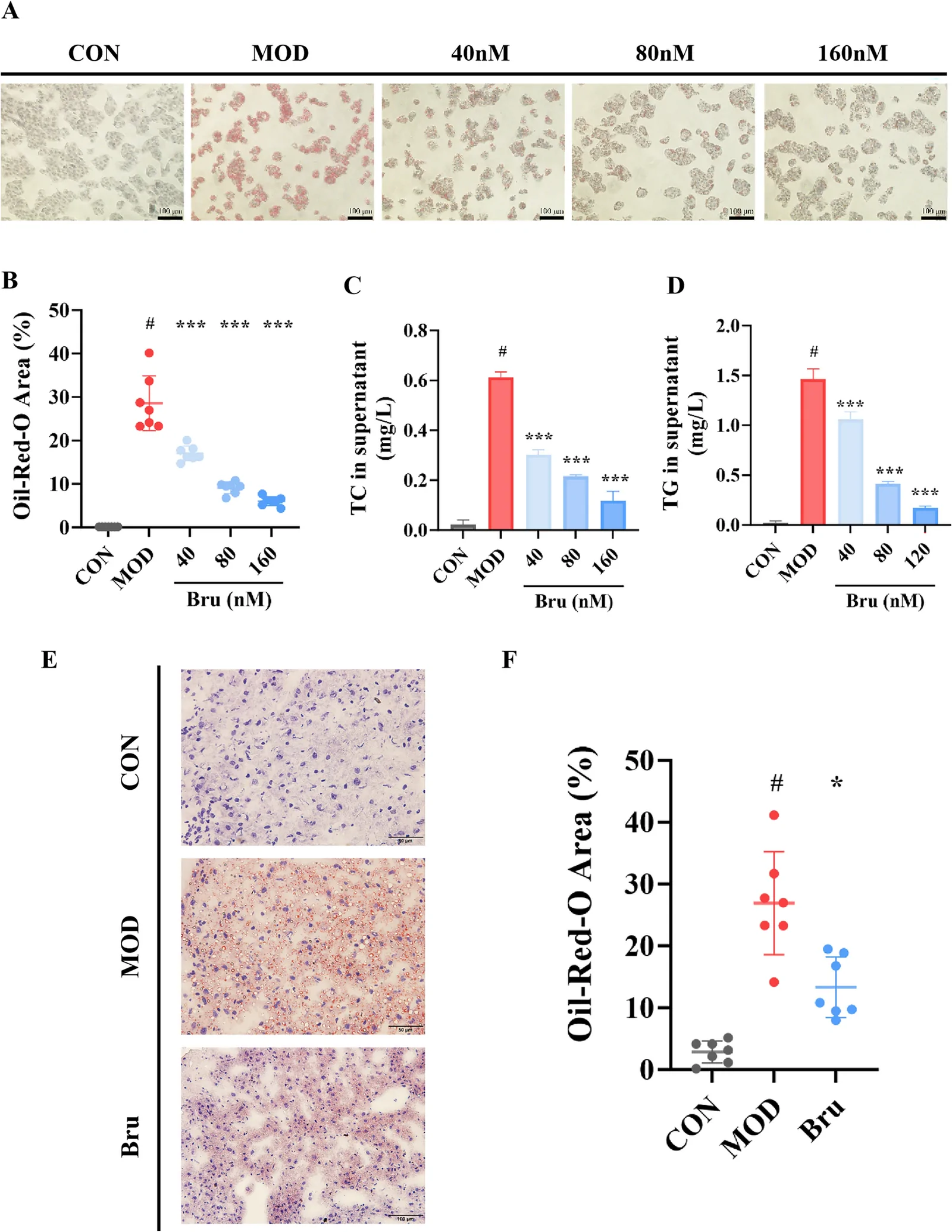
Brusatol suppresses lipid synthesis and accumulation in vitro* and *in vivo. **A** Representative Oil Red O-stained HepG2 cells induced with oleic acid (OA, 100 μM) and treated with Brusatol (40, 80, 160 nM) for 24 h. Lipid droplets are stained red; nuclei are counterstained with hematoxylin (blue). Scale bar: 50 μm. **B** Quantitative analysis of Oil Red O staining intensity in (**A**), normalized to the OA-induced model group. **C**, **D** Secreted levels of **C** total cholesterol (TC) and **D** triglycerides (TG) in the culture supernatant of HepG2 cells under the conditions described in (**A**). **E** Oil Red O staining of frozen liver sections from control (Con), IMQ-induced model (Mod), and Brusatol-treated (Bru) mice. Scale bar: 100 μm. **F** Quantification of lipid droplet area in liver sections based on (**E**). All data are expressed as mean ± SEM (n = 3 independent experiments for (**A**–**D**); n = 7 mice per group for (**F**)).***p < 0.01 versus the OA-induced (**A**–**D**) or IMQ-induced Mod (**F**) group; #*p* < 0.01 versus the untreated control group

Since keratinocytes possess limited lipid storage capacity and psoriatic dyslipidemia predominantly presents as elevated circulating glycerolipids of hepatic origin, we focused our investigation on liver-derived metabolic pathways and employed an oleic acid/palmitic acid (OA/PA)-induced steatotic HepG2 model to investigate brusatol’s lipid-modulating effects. Oil Red O staining demonstrated a significant and dose-dependent reduction in lipid droplet accumulation in HepG2 cells following brusatol treatment (40–160 nM), with maximal 57.14% clearance observed at the highest concentration (Fig. 5A, B).

Subsequently analysis of TC and TG release levels into the cell supernatant revealed that brusatol treatment markedly reduced both TC (62.3 ± 5.1% reduction, *p* < 0.001) and TG (58.7 ± 4.8% reduction, *p* < 0.001) in HepG2 cells (Fig. 5C, D), mirroring the lipid-lowering effects observed in our psoriasiform mouse model, where serum TC and TG levels were decreased by 59% and 64%, respectively (Fig. 4A).

To corroborate these findings in vivo, we performed Oil Red O staining on liver tissues obtained from the animal study. Liver sections from model group mice exhibited significantly increased lipid droplet accumulation, whereas brusatol treatment substantially reduced hepatic lipid deposition by 47% (Fig. 5E, F).

Collectively, these results demonstrate that brusatol significantly suppresses both lipid biosynthesis and release while effectively mitigating intracellular lipid accumulation, consistent with our prior lipid metabolomics findings.

### Brusatol ameliorates lipid dysregulation in psoriatic mice by targeting IL-1β

**Fig. 6:**
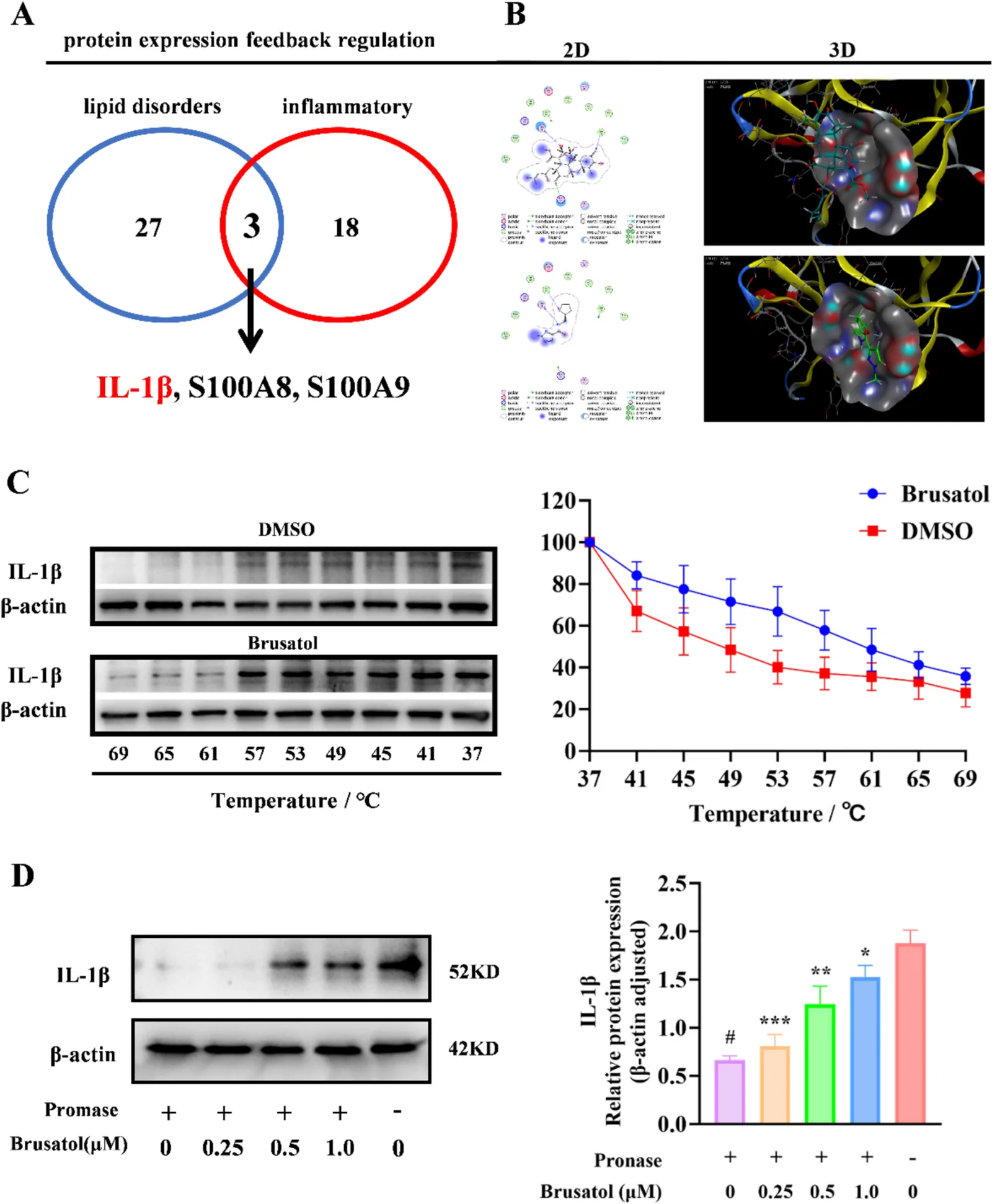
Brusatol directly binds to IL-1β and stabilizes the protein. **A** Venn diagram depicting the overlap of differentially expressed proteins involved in lipid metabolism and inflammatory response pathways from proteomic profiling, highlighting IL-1β as a central mediator. **B** Molecular docking-predicted binding conformation of Brusatol (green sticks) within the IL-1β binding pocket (PDB: 5R8Q, surface representation). Key interacting residues are indicated as cyan sticks. **C** Cellular Thermal Shift Assay (CETSA) validating the direct binding of Brusatol to IL-1β in HaCaT cell lysates. Lysates treated with DMSO or Brusatol (20 μM) were subjected to heating at graded temperatures, followed by immunoblot analysis of soluble IL-1β (upper panel). Band intensities were quantified and normalized to the 37 °C control (lower panel). **D** Drug Affinity Responsive Target Stability (DARTS) assay showing concentration-dependent stabilization of IL-1β by Brusatol. HaCaT lysates incubated with Brusatol (0 ~ 1 μM) were digested with pronase, and IL-1β levels were assessed by immunoblotting (upper panel) and quantified (lower panel). All data represent mean ± SEM; n = 3. *p < 0.05, **p < 0.01, ***p < 0.001 versus DMSO control at corresponding temperatures (**C**) or versus 0 μM Brusatol (**D**)

Both proteomic and lipidomic analyses indicated that brusatol concurrently attenuates inflammatory responses and suppresses lipogenic processes in psoriasis, suggesting its dual modulatory effects on both lipid metabolism and inflammatory pathways. To elucidate the molecular targets underlying these therapeutic effects, we performed systematic analysis of differentially expressed proteins (DEPs) identified through proteomic screening. This analysis revealed 27 lipid metabolism-associated DEPs and 18 inflammation-related DEPs. Intersectional mapping identified three overlapping DEPs, namely IL-1β, S100A8, and S100A9 (Fig. 6A). Based on existing evidence establishing IL-1β as an upstream regulator of both S100A8 and S100A9, we prioritized IL-1β as the most promising candidate for molecular docking validation.

We retrieved four high-resolution (< 2 Å) X-ray crystal structures of the IL-1β receptor complex from the PDB database and performed molecular docking simulations between brusatol and both the protein structures and their bound ligands. Analysis of three ligand-bound protein complexes revealed that brusatol effectively binds to IL-1β, with calculated binding energies ranging from − 7.01 to − 5.73 kcal/mol. These favorable energies (all below the threshold of − 5 kcal/mol) indicate strong binding affinity. Additionally, docking with the ligand-free structure (8RYS) also yielded a favorable binding energy (− 6.13 kcal/mol), reinforcing the reproducibility of this interaction. Collectively, these computational findings identify a druggable binding pocket on IL-1β that accommodates brusatol, implying a plausible mechanism by which brusatol may disrupt IL-1β-mediated signaling (Fig. 6B & Supplementary Fig.S1). The calculated binding free energies (ΔG_bind) for the top-ranked poses of brusatol with each IL-1β structure are summarized in Supplementary Table S2. To further assess the specificity of the brusatol-IL-1β interaction, we performed molecular docking of brusatol with the upstream and downstream proteins of IL-1β. The results showed binding energies greater than -5 kcal/mol in all cases, suggesting that brusatol specifically targets IL-1β. This specific binding likely contributes to the observed reduction in IL-1β levels (Supplementary Table S3).

To validate the molecular docking results and confirm the direct binding of brusatol to IL-1β, we performed cellular thermal shift assays (CETSA) and drug affinity responsive target stability (DARTS) experiments. Leveraging the high endogenous expression of IL-1β in HaCaT keratinocytes, we prepared protein lysates from this cell line for both assays. CETSA revealed that brusatol-treated samples maintained significantly higher levels of intact protein compared to the DMSO control group across increasing temperatures, with the most pronounced difference observed at 53 °C (34% higher protein stability, Fig. 6C). DARTS further demonstrated dose-dependent stabilization of IL-1β against enzymatic degradation in the presence of brusatol, with markedly increased IL-1β protein levels correlating with higher brusatol concentrations (Fig. 6D). These findings conclusively demonstrate direct binding between brusatol and IL-1β, strongly supporting our in-silico predictions.

**Fig. 7:**
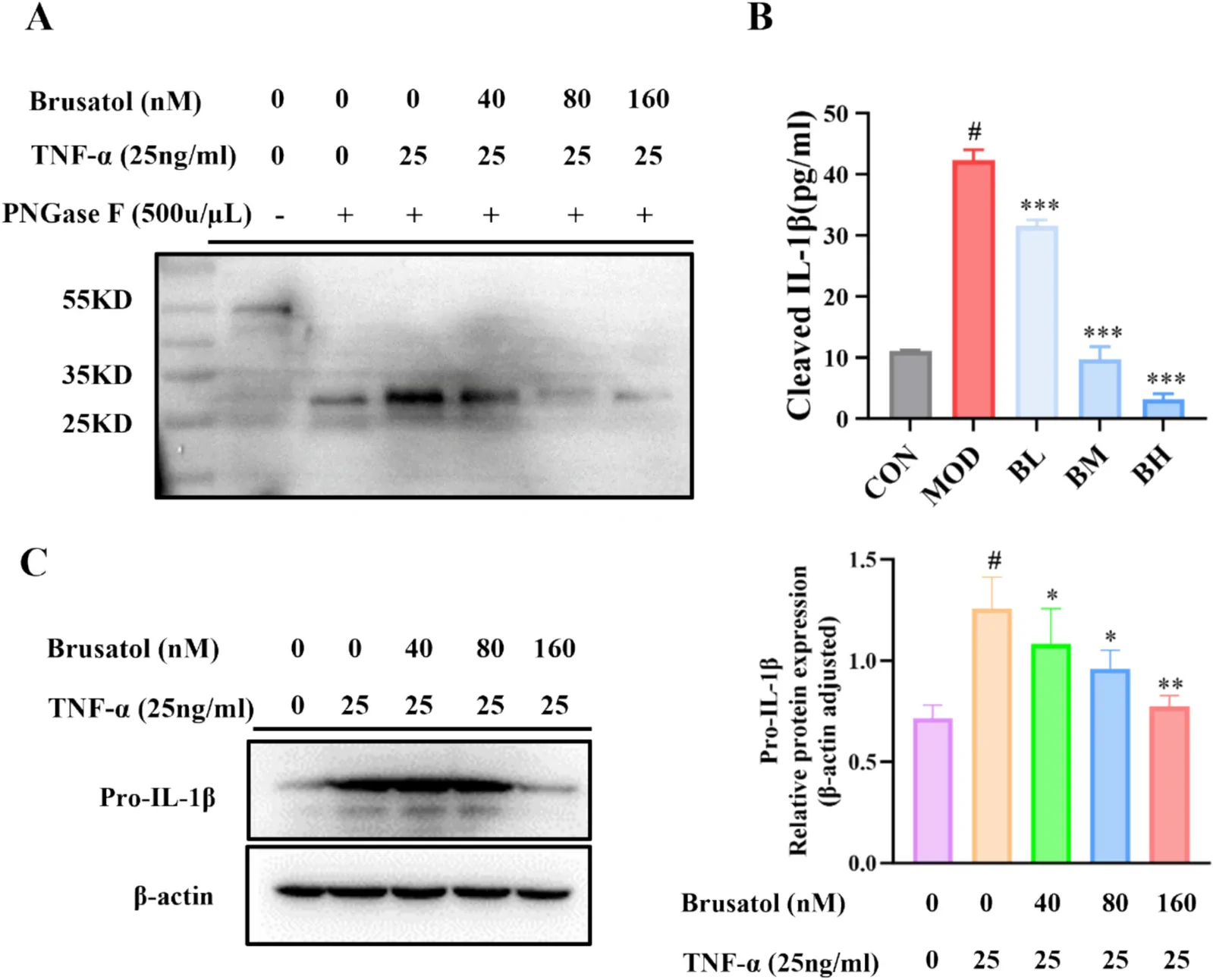
Brusatol reduces both pro-IL-1β and mature IL-1β levels in HaCaT keratinocytes. **A** PNGase F deglycosylation assay identifies the ~ 52 kDa band detected in CETSA/DARTS as pro-IL-1β. Cell lysates from TNF-α (25 ng/mL)-stimulated HaCaT cells treated with brusatol (0 ~ 160 nM) were digested with PNGase F, resulting in a mobility shift from ~ 52 kDa to ~ 31 kDa, consistent with the unglycosylated pro-IL-1β precursor. **B** Brusatol dose-dependently inhibits the secretion of mature IL-1β. The levels of cleaved IL-1β in cell culture supernatants was measured by ELISA. Data are mean ± SEM (n = 3). 0**C** Brusatol decreases intracellular pro-IL-1β protein expression. Representative Western blot and corresponding quantification show decreased pro-IL-1β expression in cell lysates following brusatol treatment. Data were normalized to β-actin and are presented as mean ± SEM (n = 3). *p < 0.05, **p < 0.01 versus TNF-α-stimulated control. #*p*<0.05 versus untreated control group.

Notably, the detected protein exhibited a molecular weight of approximately 52 kDa, which is inconsistent with the mature IL-1β cytokine (17 kDa) and ruling out the possibility that the detected band corresponds to the cleaved form. To definitively clarify the nature of the 52 kDa band and brusatol’s action, we conducted targeted experiments. Deglycosylation with PNGase F confirmed the band corresponds to the glycosylated, inactive precursor of IL-1β (pro-IL-1β), as evidenced by its migration shift to ~ 31 kDa upon treatment (Fig. 7A). Subsequent quantification of both IL-1β forms revealed that brusatol dose-dependently suppressed both TNF-α-induced secretion of mature IL-1β (measured by ELISA; Fig. 7B) and intracellular levels of pro-IL-1β (assessed by Western blot; Fig. 7C). Collectively, these findings demonstrate that brusatol does not directly target the mature cytokine. Instead, it likely binds to and stabilizes pro-IL-1β upstream (as initially suggested by CETSA/DARTS for the 52 kDa species), yet this interaction ultimately results in a reduction in its cellular abundance, consequently limits the precursor pool available for proteolytic cleavage into the mature, bioactive form.

**Fig. 8:**
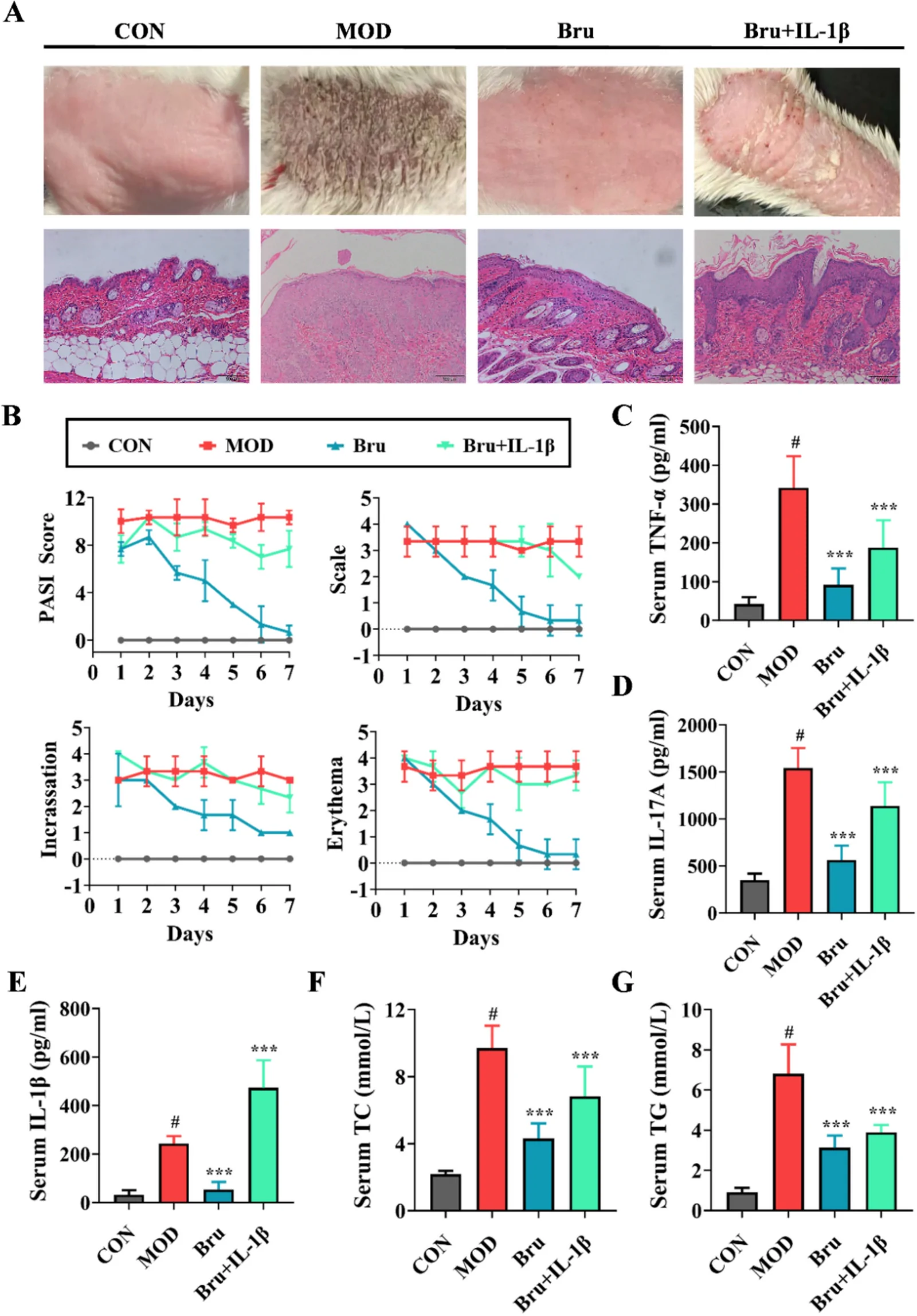
Exogenous IL-1β administration reverses the therapeutic effects of brusatol in IMQ-induced psoriatic mice. **A** Representative clinical photographs of dorsal skin (upper panel) and corresponding H&E-stained histological sections (lower panel) from mice at the endpoint of the rescue experiment. Mice received Brusatol (Bru, 4.6 mg/kg) alone or in combination with recombinant murine IL-1β (10 mg/kg, intradermally injected into lesional skin). Scale bar: 100 μm. **B** Time-course changes in the total Psoriasis Area and Severity Index (PASI) score over the 7-day treatment period across the indicated groups. **C**, **E** Serum concentrations of IL-1β (**C**), IL-17A (**D**), and TNF-α (**E**) measured by ELISA following the intervention. **F**, **G** Post-intervention serum levels of **F** total cholesterol (TC, **F**) and triglycerides (TG, **G**). Data in (**B**–**G**) are expressed as mean ± SD (n = 8). ***p < 0.001 for Bru + IL-1β.Bru alone *vs. *IMQ-induced model group; ^#^*p* < 0.05 for IMQ-induced model group *vs*. control group.

To further validate that brusatol exerts its therapeutic effects by targeting IL-1β, we conducted a loss-of-function rescue paradigm in mice with IMQ-induced psoriasiform dermatitis. During the intervention phase, cohorts received either: (i) the standard oral brusatol regimen ( 4.6 mg/kg/day), or (ii) identical brusatol dosing combined with daily intradermal injections of recombinant murine IL-1β (10 mg/kg) administered topographically to lesional dermatomes. This combinatorial strategy aimed to compensate for brusatol-mediated IL-1β suppression. Visual inspection of skin lesions demonstrated incomplete resolution in the IL-1β-supplemented group, contrasting sharply with the marked improvement observed in the standard treatment group (brusatol monotherapy). Histopathological evaluation further revealed that mice receiving supplemental IL-1β exhibited sustained hyperkeratosis, acanthosis, and prominent perivascular inflammatory cell infiltration in the dermis compared to the brusatol-only group (Fig. 8A). Consistent with these findings, PASI scoring quantified 50–150% elevations across all disease progression metrics in the IL-1β rescue group versus controls, including erythema intensity (*p* = 0.008), scaling severity (*p* = 0.003), and lesional induration (*p* < 0.001) (Fig. 8B).

Serum cytokine analysis in mice demonstrated that IL-1β supplementation during treatment attenuated the anti-inflammatory efficacy of brusatol, leading to elevated levels of IL-17A, TNF-α, and IL-1β. Notably, IL-1β exhibited the most pronounced increase, likely attributable to the exogenous administration (Fig. 8C, E). Mirroring the inflammatory profile, serum lipid analysis revealed a partial reversal of brusatol’s hypolipidemic effects. The most substantial attenuation was observed in TC regulation, with a 43% reduction in treatment efficacy compared to brusatol monotherapy. In contrast, TG levels demonstrated relative resistance to this effect, maintaining 92% of the therapeutic response (Fig. 8F, G).

Collectively, these results demonstrate that IL-1β effectively counteracts the therapeutic effects of brusatol in psoriatic mice, providing compelling evidence that brusatol exerts its biological activity primarily through IL-1β targeting.

### Specific interaction between IL-1β and AMPK

While our previous findings established IL-1β as the direct target of brusatol, the precise mechanism by which IL-1β regulates lipid metabolism remained unclear. To elucidate this pathway, we first generated IL-1β-overexpressing HaCaT cells models and then treated them with a safe dose (5 nM) of the Gevokizumab (a monoclonal antibody specifically targeting IL-1β), subsequently analyzing changes in key lipid pathway proteins.

**Fig. 9:**
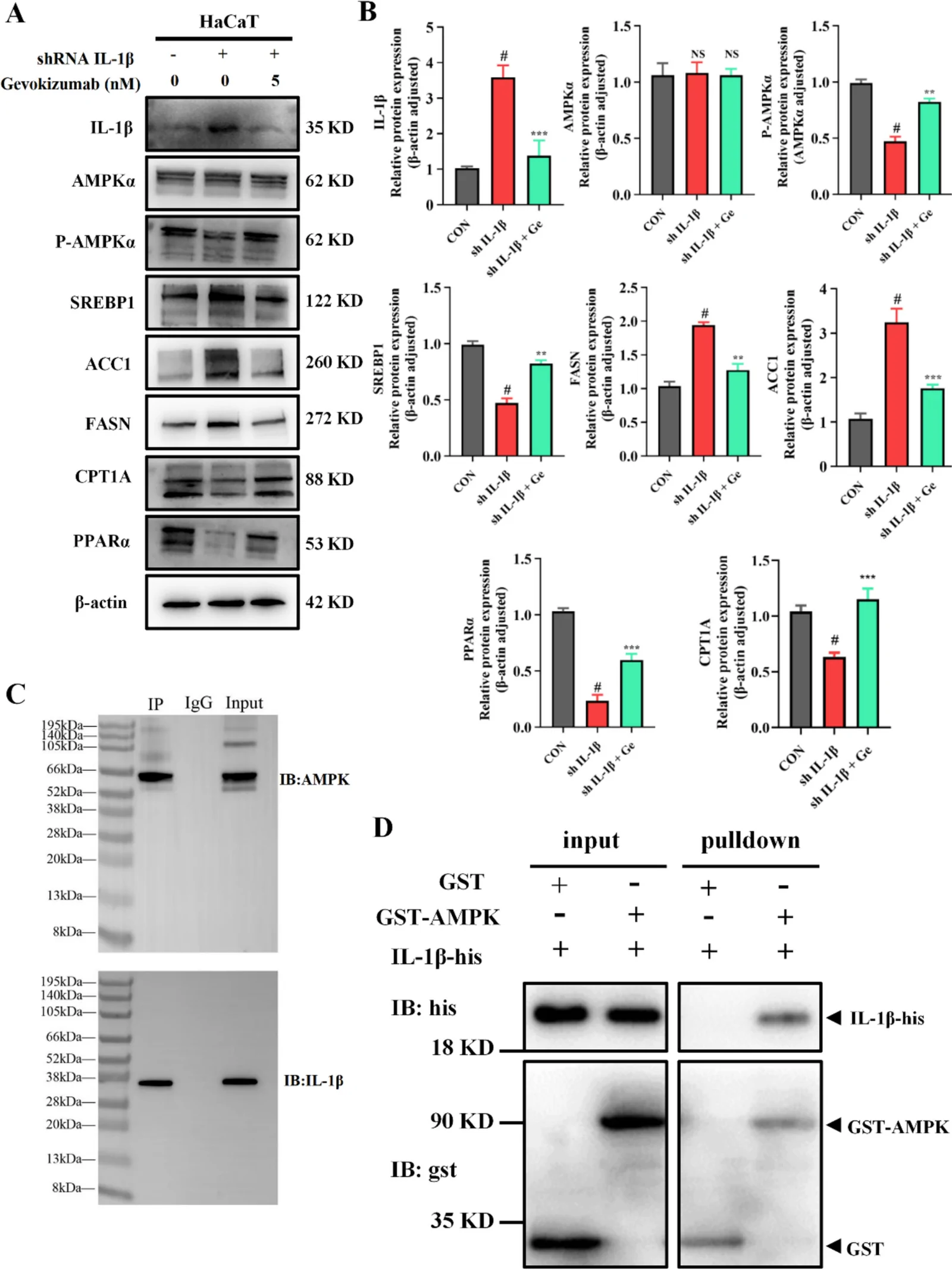
Direct interaction of IL-1β with AMPK and its functional antagonism. **A**, **B** The impact of IL-1β manipulation on the AMPK signaling pathway was evaluated in HaCaT cells. Following IL-1β overexpression (24 h) and subsequent neutralization with Gevokizumab (5 nM, 12 h), protein levels of AMPKα and its downstream lipid metabolic targets were analyzed by immunoblotting (**A**) and quantified (**B**). β-actin was used as a loading control. **C** Co-immunoprecipitation (Co-IP) suggests an association between IL-1β and AMPK in HaCaT cell lysates. Whole-cell lysates were immunoprecipitated (IP) with an anti-IL-1β antibody or control IgG, followed by immunoblotting (IB) with anti-AMPKα and anti-IL-1β antibodies. Input represents 10% of the total lysate used for IP. **D** A direct interaction between AMPK and IL-1β was confirmed by an in vitro GST pull-down assay. Purified His-tagged IL-1β was incubated with GST or GST-AMPK fusion protein immobilized on glutathione sepharose beads. Bound proteins were eluted and detected by immunoblotting using anti-His and anti-GST antibodies. Quantitative data are presented as mean ± SEM from three independent experiments.**p < 0.01, ***p < 0.001 compared to the Mock group; ^#^*p* < 0.05 compared to the IL-1β OE group

Intriguingly, IL-1β overexpression demonstrated a pronounced propensity for lipid biosynthesis while significantly suppressing fatty acid β-oxidation pathways, potentially mediated through inhibition of the metabolic regulator AMPK (a central lipid metabolic switch) (Fig. 9A, B). Subsequent gevokizumab intervention effectively reversed this phenotype, markedly suppressing upregulated lipogenic proteins while reactivating β-oxidation pathway components.

The highly coordinated changes in these lipid-associated proteins suggested that IL-1β likely exerts its regulatory effect by modulating AMPK, the master upstream switch in the lipid pathway. To explore a potential interaction between IL-1β and AMPK, we first performed co-immunoprecipitation (Co-IP) assays using HaCaT cell lysates. Immunoprecipitation with an anti-IL-1β antibody successfully co-precipitated AMPK, whereas control IgG did not (Fig. 9C), suggesting a potential association between IL-1β and AMPK in a cellular context. However, as Co-IP cannot distinguish between direct and indirect interactions, we next sought to obtain definitive evidence for a direct physical binding.

Therefore, we conducted an in vitro GST pull-down assay using purified recombinant proteins to eliminate the possibility of intermediary factors. Purified GST-tagged AMPK specifically pulled down His-tagged IL-1β, while GST alone did not (Fig. 9D).

This result, obtained in a cell-free system, conclusively establishes a direct physical interaction between IL-1β and AMPK, independent of other cellular factors.

### Brusatol restores psoriatic skin lipid dysregulation through IL-1β–dependent AMPK activation

**Fig. 10:**
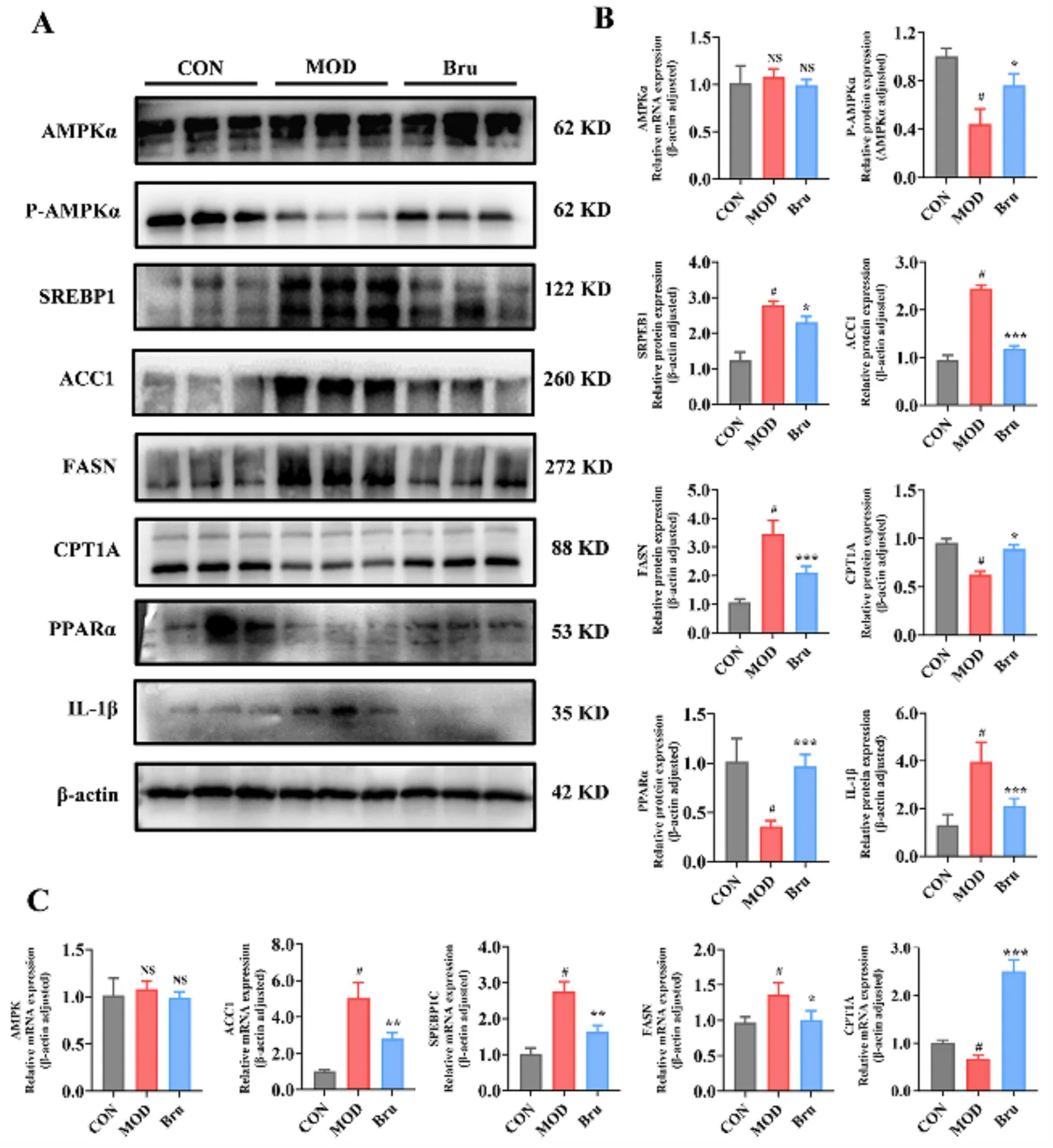
Brusatol alleviates lipid dysregulation in IMQ-induced psoriatic mice by targeting IL-1β and subsequently activating the AMPK signaling pathway. **A** Representative western blots and **B** quantitative analysis of key proteins in the AMPK pathway in dorsal skin tissues from control (Con), model (Mod), and Brusatol-treated (Bru, 4.6 mg/kg) mice. Protein levels of total AMPKα, phosphorylated AMPKα (p-AMPKα), and downstream effectors for fatty acid oxidation (PPARα, CPT1A) and lipogenesis (SREBP-1, FASN, ACC1) were assessed, with β-actin as a loading control. **C** mRNA expression levels of *Srebp-1c*, *Acc1*, *Fasn*, *Pparα*, and *Cpt1a* in dorsal skin tissues, as determined by RT-qPCR. Data in **B** and **C** are presented as mean ± SEM (n = 9 for (**B**); n = 5 for (**C**)). *p < 0.05, **p < 0.01, ***p < 0.001 *vs*. Mod group. #*p*<0.05* vs.* control group.

Subsequently, we validated these findings at the animal level. Our results demonstrated that brusatol enhances AMPK phosphorylation and stimulates fatty acid β-oxidation protein synthesis by suppressing IL-1β expression. This cascade of events resulted in significant upregulation of both protein and RNA levels of the downstream effectors PPARα and CPT1A. Moreover, the increase in phosphorylated AMPK effectively inhibited the activation of the lipogenic pathway. As a consequence, both protein and RNA expression levels of key lipogenic regulators (SREBP1, FASN, and ACC1) were substantially downregulated, ultimately ameliorating the metabolic dysregulation associated with psoriasis (Fig. 10).

## Discussion

Our study delineates a novel pharmacological strategy for ameliorating psoriatic dyslipidemia by targeting the inflammation-metabolism interplay. We demonstrate that the natural quassinoid brusatol effectively alleviates both cutaneous inflammation and systemic lipid dysregulation in an IMQ-induced murine model of psoriasis. Through integrated proteomic and lipidomic profiling, we identified IL-1β as a key node linking psoriatic inflammation to lipid metabolic dysfunction. A series of mechanistic studies, encompassing molecular docking, CETSA, DARTS, and rescue experiments, unequivocally established IL-1β as a direct functional target of brusatol. Most importantly, we uncovered a previously unrecognized physical interaction between IL-1β and AMPK, whereby IL-1β sequesters AMPK in the cytoplasm, thus inhibiting its activity. Brusatol, by binding to IL-1β, disrupts this complex, facilitating AMPK nuclear translocation and subsequently restoring lipid homeostasis by downregulating the SREBP-1c/FASN/ACC1 lipogenic axis and upregulating the PPARα/CPT1A fatty acid β-oxidation pathway. These findings not only position brusatol as a promising multi-target agent for psoriasis and its comorbidities but also reveal a fundamental IL-1β/AMPK regulatory axis in inflammation-metabolism crosstalk.

As research progresses, psoriasis is increasingly recognized as a systemic inflammatory condition with multi-organ involvement, rather than a localized dermatosis. Systemic manifestations of psoriasis prominently affect musculoskeletal (particularly arthritis), cardiovascular, neuropsychiatric, and metabolic systems. Of these, metabolic dysregulation, particularly lipid metabolism disorders characterized by hypercholesterolemia and hypertriglyceridemia, shows high comorbidity prevalence [3]. A retrospective study corroborated this, revealing significantly higher serum TC and low-density lipoprotein cholesterol (LDL-C) levels in patients with moderate-to-severe psoriasis compared to healthy controls [18]. This dyslipidemia may be mediated by inflammatory cytokines (particularly TNF-α) through suppression of lipoprotein lipase (LPL) activity [19]. Mechanistically, LPL inhibition reduces triglyceride catabolism while enhancing hepatic very-low-density lipoprotein (VLDL) synthesis, consequently elevating circulating TC and LDL-C levels [20]. Notably, our in vivo and in vitro experiments consistently demonstrated pronounced lipid accumulation in psoriatic models, corroborating these clinical observations.

The interplay between dyslipidemia and chronic inflammation is a cornerstone of psoriatic systemic comorbidity, forming a self-perpetuating cycle that our findings help to elucidate. Rather than parallel pathways, these processes are mechanistically intertwined. Pro-inflammatory cytokines such as IL-1β, TNF-α, and IL-17 disrupt systemic lipid homeostasis by suppressing lipoprotein lipase and promoting hepatic lipogenesis, leading to hyperlipidemia [21, 22]. Conversely, lipid abnormalities actively sustain inflammation—for instance, cholesterol enrichment facilitates inflammatory receptor signaling via lipid raft assembly [23], and oxidized LDL acts as a damage-associated molecular pattern to perpetuate immune activation [24]. Moreover, dysregulated lipid metabolism underpins the differentiation of pathogenic Th17 cells, key drivers of psoriasis [25]. Thus, brusatol’s amelioration of lipid metabolism is not an epiphenomenon but a central therapeutic event. By targeting IL-1β and restoring AMPK-mediated lipid homeostasis, brusatol disrupts this pathogenic circuit, depriving inflammation of its metabolic fuel and promoting sustained resolution.

The management of psoriasis has now entered the biologics era. Undeniably, IL-17 inhibitors have revolutionized treatment outcomes, elevating therapeutic benchmarks from PASI75 to PASI90 response criteria. However, accumulating clinical data indicate that extended IL-17 inhibitor therapy leads to gradually increasing rates of drug resistance and off-target effects. Treatment-resistant cases frequently exhibit compensatory activation of either the Th1 pathway or IL-23 signaling cascade [26]. This phenomenon illustrates the inherent limitations of highly specific biologic therapies. Concurrently, while existing biologics effectively ameliorate cutaneous manifestations, they demonstrate limited efficacy in improving concomitant metabolic disturbances such as dyslipidemia or insulin resistance [27]. This therapeutic gap underscores the urgent need to develop novel agents with dual anti-inflammatory and metabolic regulatory properties.

Emerging evidence suggests that brusatol exerts multi-target effects by concurrently inhibiting the inflammatory pathways (e.g., NF-κB, MAPK, and NLRP3) while modulating the AMPK-Nrf2 metabolic axis, thereby overcoming the limitations and drug resistance associated with single-target therapies [28–31]. Our lipidomics analysis further revealed that brusatol significantly reduced serum diacylglycerol (DAG) levels in a psoriasiform dermatitis mouse model. Subsequent in vivo experiments demonstrated its potent regulatory effects on key metabolic pathways, including the AMPK/PPARα/CPT1A axis (promoting fatty acid oxidation) and the SREBP1/FASN/ACC1 axis (suppressing lipogenesis). This dual modulation of inflammatory and metabolic circuitry distinguishes brusatol from conventional biologics, positioning it as a promising therapeutic for psoriasis-metabolic syndrome comorbidity.

Our proteomic analysis revealed significant alterations in inflammatory markers following brusatol treatment, with IL-1β emerging as the inflammatory factor most closely linked to lipid dysregulation. As a pivotal initiator of inflammation, IL-1β is predominantly secreted by keratinocytes, macrophages, and dendritic cells [32]. In vitro experiments confirmed that brusatol effectively suppresses IL-1β release from keratinocytes. Notably, epidermal keratinocytes in IMQ-treated mice exhibit minimal release IL-1β due to interspecies differences [33]. Instead, macrophages and dendritic cells serve as the predominant sources. Our in vivo findings demonstrated that oral brusatol administration significantly reduced IL-1β levels in mouse skin, suggesting that brusatol also suppresses IL-1β expression in immune cells such as macrophages and dendritic cells. Consistent with these findings, our in vivo studies revealed that brusatol administration via oral gavage induced a 50% suppression of IL-1β in cutaneous tissues (*p* < 0.01), suggesting its additional suppressive effect on IL-1β expression in immune cells, particularly macrophages and dendritic cells.

Studies have demonstrated that mature IL-1β, cleaved by caspase-1, activates the IL-1R signaling pathway, promoting the proliferation of dermal γδT17 cells and synergizing with IL-23 to enhance IL-17 production [34–36]. Our multi-omics analysis uncovered pathway-specific regulation, where IL-1R accessory protein (IL-1RAcP) exhibited a change concordant with IL-1β, while the IL-1 receptor antagonist (IL-1Ra) showed an inverse trend. This pattern aligns with the known physiological function of the IL-1R pathway under inflammation conditions. Strikingly, caspase-1 levels were unperturbed upon brusatol treatment, suggesting that brusatol-mediated suppression of IL-1β is independent of caspase-1 processing. Instead, brusatol likely directly targets IL-1β to suppress its expression. This hypothesis was further corroborated by subsequent molecular docking and CETSA, which demonstrated a direct physical interaction between brusatol and IL-1β.

Beyond its inflammatory roles, IL-1β exhibits particularly intriguing temporally biphasic regulation regulatory role in lipid metabolism, exhibiting short-term stimulatory but long-term inhibitory effects. Emerging evidence demonstrates that IL-1β can fully restore adipogenic capacity in human adipose-derived stem cells (hASCs) under suboptimal differentiation conditions (without 3-isobutyl-1-methylxanthine) within 2 h via rapid activation of the p38-CREB-C/EBPδ/β axis. Paradoxically, chronic IL-1β exposure leads to sustained NF-κB activation, which subsequently suppresses PPARγ and C/EBPα expression, ultimately inhibiting adipogenesis and inducing insulin resistance [37, 38]. This dual-phase regulation may explain the clinical observation of psoriasis patients developing type 2 diabetes, as persistently elevated IL-1β levels are characteristic of psoriatic inflammation [39, 40]. The transient lipid abnormalities detected in our murine model may relate to the relatively short experimental duration, highlighting the importance of investigating dynamic lipid changes in early-stage psoriasis patients, as these metabolic perturbations may precede overt metabolic disease development.

As a cellular energy sensor, AMP-activated protein kinase (AMPK) orchestrates diverse biological processes including metabolism, inflammation, and autophagy through phosphorylation-mediated activation of downstream targets [41]. Emerging evidence indicates that primary keratinocytes derived from psoriatic lesions exhibit a "Warburg-like" metabolic phenotype characterized by enhanced glycolysis and elevated reactive oxygen species (ROS) production [42]. Notably, AMPK activation can suppress the mTOR-HIF-1α axis, subsequently downregulating glycolytic rate-limiting enzymes (HK2 and PKM2) and reducing ROS generation, thereby ameliorating metabolic reprogramming. Regarding the autophagy-inflammation axis, current research has revealed significant suppression of the AMPK → ULK1(S317/S777) → Atg7 pathway in psoriatic models, leading to impaired clearance of inflammasome components and damage-associated molecular patterns (e.g., S100A8/A9) [43–45]. This pathological cascade was effectively reversed by administration of the AMPK agonist acadesine in IMQ-induced murine models. Our proteomic findings corroborate these observations, demonstrating that brusatol treatment significantly reduce the expression of pro-inflammatory mediators (S100A8, S100A9) and the NF-κB inhibitor IκB in psoriatic mouse skin, further implicating AMPK activation as a key mechanistic component of brusatol’s therapeutic mechanism, bridging metabolic regulation and inflammatory resolution.

A key finding from our safety assessment is that brusatol significantly lowers serum TG and TC levels even in healthy, normolipidemic mice. This effect, consistent with its efficacy in reversing IMQ-induced dyslipidemia, suggests its lipid-modulating action extends beyond a mere anti-inflammatory bystander effect, instead reflecting an intrinsic pharmacological property. The evidence indicates that brusatol regulates AMPK-mediated lipid homeostasis across both physiological and pathological states. This broad-spectrum efficacy underscores its therapeutic promise but also necessitates careful lipid profiling in future clinical applications to avoid over-reduction.

 Current research indicates that AMPK indirectly suppresses IL-1β activity through multiple signaling pathways. In an obesity-related study, AMPK phosphorylates the SUCLA2 metabolic enzyme to restrict glutaminolysis, thereby reducing succinate accumulation and blocking NLRP3 inflammasome activation, which ultimately attenuates pro-IL-1β transcription [46]. Additional research demonstrated that AMPK activation suppresses IL-1β-induced IRAK4-MKK4/JNK and IKK/IκB/NF-κB signaling cascades in adipocytes, thereby suppressing the secretion of chemokines such as CXCL10 [47].

To directly validate the regulatory interplay between AMPK and IL-1β, we conducted IL-1β overexpression in HaCaT cells. Results revealed that total AMPK expression remained unaltered, whereas phosphorylated AMPK (pAMPK) levels were significantly suppressed with increasing IL-1β expression. This phenomenon was reversed upon addition of the IL-1β inhibitor Gevokizumab, which restored pAMPK expression. Collectively, these data demonstrate that IL-1β impedes AMPK nuclear translocation by disrupting its nuclear import machinery, thereby trapping AMPK within the cytoplasmic compartment. This intriguing phenomenon evokes an analogy to the canonical NF-κB/IκB regulatory paradigm, wherein IκB sequesters NF-κB within the cytoplasmic compartment through direct steric hindrance, thereby effectively blocking its nuclear translocation [48, 49]. To systematically interrogate whether AMPK and IL-1β employ a cognate regulatory mechanism, we performed co-immunoprecipitation (CoIP) assays. The results revealed a specific direct physical interaction between AMPK and IL-1β. However, the underlying molecular mechanisms governing this interaction remain elusive and warrant further investigation.

Despite these compelling findings, several limitations of our study should be acknowledged. First, while we demonstrate a central role for the IL-1β–AMPK axis, the relative contributions of different cellular sources of IL-1β (e.g., keratinocytes, macrophages) and different tissue compartments (e.g., skin vs. liver) to the overall psoriatic dyslipidemic phenotype remain to be fully dissected. Second, although our rescue experiment with exogenous IL-1β strongly supports its role as a key target, the in vivo efficacy of brusatol might also involve other cell types or inflammatory pathways. Future studies employing cell-specific or inducible IL-1β knockout models would be invaluable to precisely delineate the mechanistic contributions of IL-1β signaling in various tissues to the pathogenesis of psoriatic dyslipidemia and the therapeutic effects of brusatol. Furthermore, investigating the pharmacokinetic profile and optimizing the delivery formulation of brusatol are crucial next steps for its translational development. Notwithstanding these limitations, our work provides a solid foundation for targeting the IL-1β–AMPK interaction as a viable therapeutic strategy for psoriasis and its associated metabolic comorbidities.

**Fig. 11:**
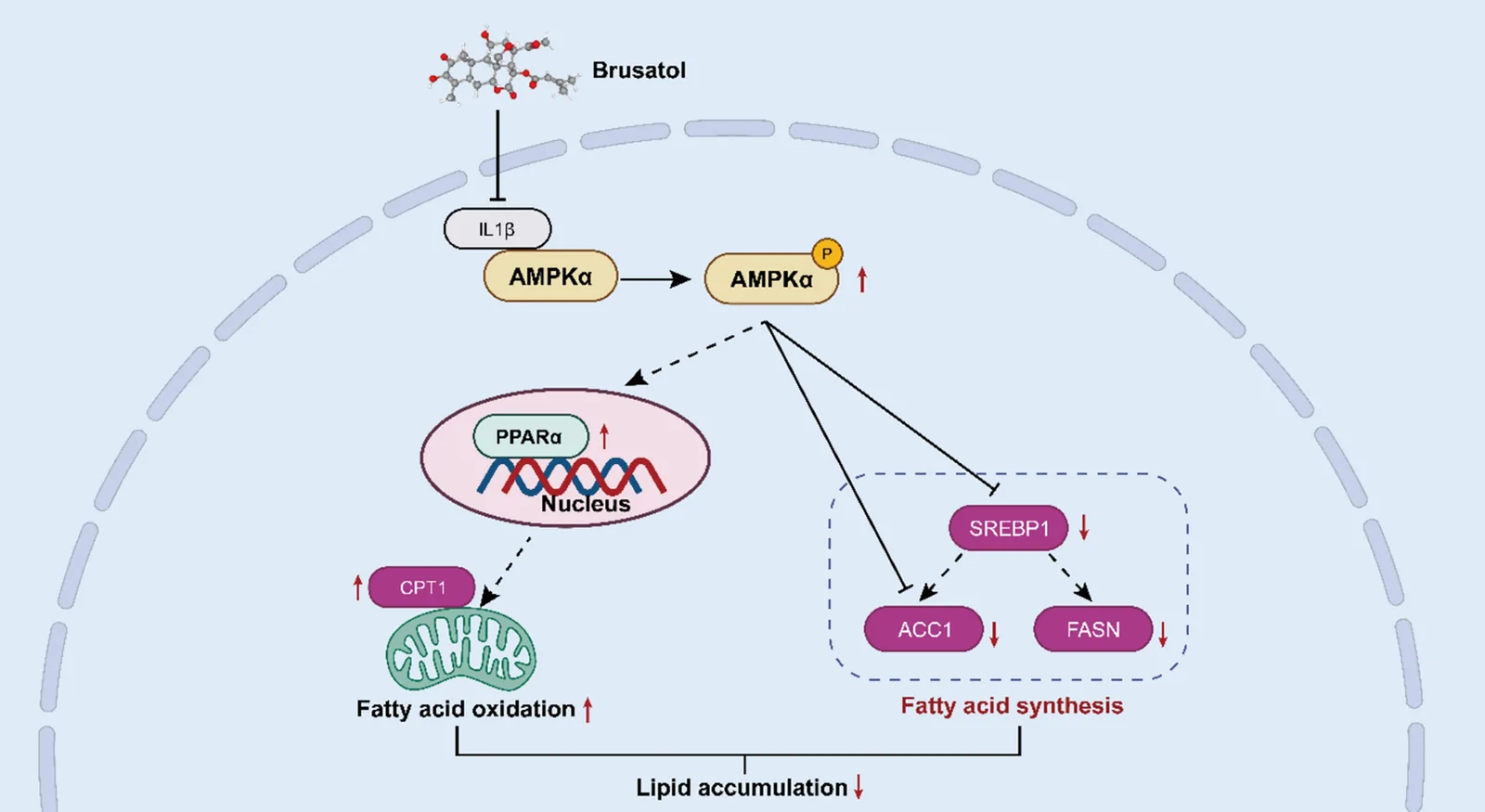
Schematic description of brusatol restores AMPK-mediated lipid homeostasis by targeting IL-1β in psoriatic dyslipidemia

Collectively, our findings demonstrate that brusatol exhibits dual anti-inflammatory andlipid-lowering efficacy in psoriasis, offering a novel therapeutic strategy for managingpsoriasis-related comorbidities. Building upon prior clinical observations [1, 2], our study specifically addressed systemic dyslipidemia associated with psoriasis, as evidenced by the marked alterations in serum lipid profiles (Fig. 4A) and perturbations in hepatic lipidmetabolism (Fig. 5E, F), rather than localized subcutaneous adipose tissue remodeling, aphenomenon with limited clinical substantiation [1, 2]. Beyond efficacy, we mechanistically identify IL-1β as a direct target of brusatol and reveal a novel cytoplasmic IL-1β-AMPK interaction that underlies inflammatory-metabolic crosstalk (Fig. 11). Brusatol’s disruption of this complex facilitates AMPK nuclear translocation and transcriptional reprogramming, elucidating a new pharmacologic mechanism and nominating the IL-1β-AMPK axis as a targetable pathway in inflammatory diseases with metabolic components.

## Supplementary Information


Supplementary material 1. Supplementary material 2. 

## Data Availability

No datasets were generated or analysed during the current study.

## Abbreviations

ACC1: Acetyl-CoA carboxylase 1
ALT: Alanine aminotransferase
AMPK: AMP-activated protein kinase
AST: Aspartate aminotransferase
BMECs: Brain microvascular endothelial cells
Caspase-1: Cysteine-aspartic protease-1
CETSA: Cellular thermal shift assay
Co-IP: Co-immunoprecipitation
CPT1A: Carnitine palmitoyl transferase 1A
CRE: Creatinine
DAG: Diacylglycerol
DARTS: Drug affinity responsive target stability
EBP: Enhancer binding protein
ELISA: Enzyme-linked immunosorbent assay
FASN: Fatty acid synthase
FASP: Filter-aided sample preparation
FBS: Fetal bovine serum
H&E: Hematoxylin and eosin
IL-17A: Interleukin-17A
IL-1Ra: IL-1 receptor antagonist
IL-1RAcP: IL-1R accessory protein
IL-1β: Interleukin-1 beta
IL-23: Interleukin-23
IMQ: Imiquimod
IκB: Inhibitor of κB
K17: Keratin 17 cholesterol
LPL: Lipoprotein lipase
LDL-C: Low-density lipoprotein
MAPK: Mitogen-activated protein kinase
MTX: Methotrexate
NF-κB: Nuclear factor kappa-light-chain-enhancer of activated B cells
NLRP3: NOD-like receptor family pyrin domain containing 3
Nrf2: Nuclear factor erythroid 2-related factor 2
OA: Oleic acid
pAMPK: Phosphorylated AMPK
PASI: Psoriasis area and severity index
PCA: Principal component analysis
PPARα: Peroxisome proliferator-activated receptor alpha
QC: Quality control
ROS: Reactive oxygen species
RT-qPCR: Real-time fluorescence quantitative technology
SREBP-1c: Sterol regulatory element binding protein 1c
TC: Total cholesterol
TEER: Trans-endothelial electrical resistance
TG: Triglycerides
Th17: T helper 17 cells
TNF-α: Tumor necrosis factor-alpha
VLDL: Very low density lipoprotein
γδT17: Gamma-delta T cell type 17
