# Correction: Brusatol ameliorates psoriatic dyslipidemia by targeting IL-1β to restore AMPK-mediated lipid homeostasis

**DOI:** 10.1186/s13020-026-01493-y

**Published:** 2026-08-28

**Authors:** Yuankuan Jiang, Shumeng Zhang, Hewen Guan, Kejia lv, Jinchao Yu, Siyi Li, Renchuan Jia, Xiujie Zhang, Shurong Ma, Jialin Qu, Jingrong Lin

**Affiliations:** 1 Laboratory of Integrative Medicine, The First Affiliated Hospital of Dalian Medical University, No. 222, Zhongshan Road, Dalian, 116011 China; 2 Department of Dermatology, The First Affiliated Hospital of Dalian Medical University, No. 222, Zhongshan Road, Dalian, 116011 China

**Correction** **:** **Chinese Medicine (2026) 21:18** **https://doi.org/10.1186/s13020-025-01287-8**

Following publication of the original article [1], the authors reported that the representative Oil Red O-stained images of HepG2 cells treated with brusatol (40 nM and 80 nM) in Fig. 5A were inadvertently duplicated.

The correct Fig. 5 has been provided in this Correction. All other panels, data, interpretations and conclusions of the manuscript remain completely unchanged.

The incorrect Fig. 5 is:

**Fig. 5:**
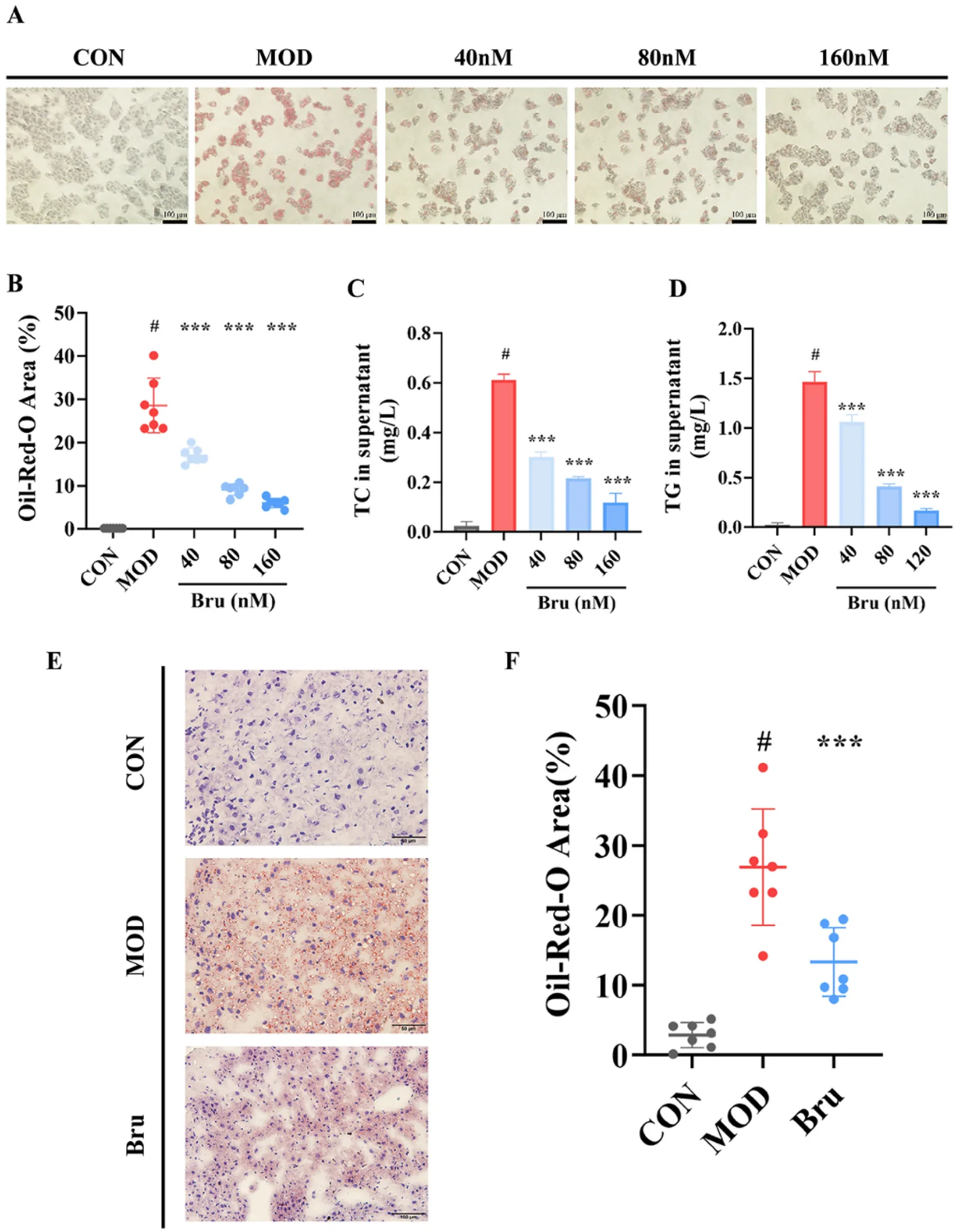
Brusatol suppresses lipid synthesis and accumulation in vitro and in vivo. **A** Representative Oil Red O-stained HepG2 cells induced with oleic acid (OA, 100 μM) and treated with Brusatol (40, 80, 160 nM) for 24 h. Lipid droplets are stained red; nuclei are counterstained with hematoxylin (blue). Scale bar: 50 μm. **B** Quantitative analysis of Oil Red O staining intensity in (A), normalized to the OA-induced model group. **C, D** Secreted levels of **C** total cholesterol (TC) and **D** triglycerides (TG) in the culture supernatant of HepG2 cells under the conditions described in (**A**). **E** Oil Red O staining of frozen liver sections from control (Con), IMQ-induced model (Mod), and Brusatol-treated (Bru) mice. Scale bar: 100 μm. **F** Quantification of lipid droplet area in liver sections based on (**E**). All data are expressed as mean ± SEM (n = 3 independent experiments for (**A**–**D**); n = 7 mice per group for (**F**)). ***p < 0.01 versus the OA-induced (**A**–**D**) or IMQ-induced Mod (**F**) group; #*p* < 0.01 versus the untreated control group

The correct Fig. 5 is:

**Fig. 5:**
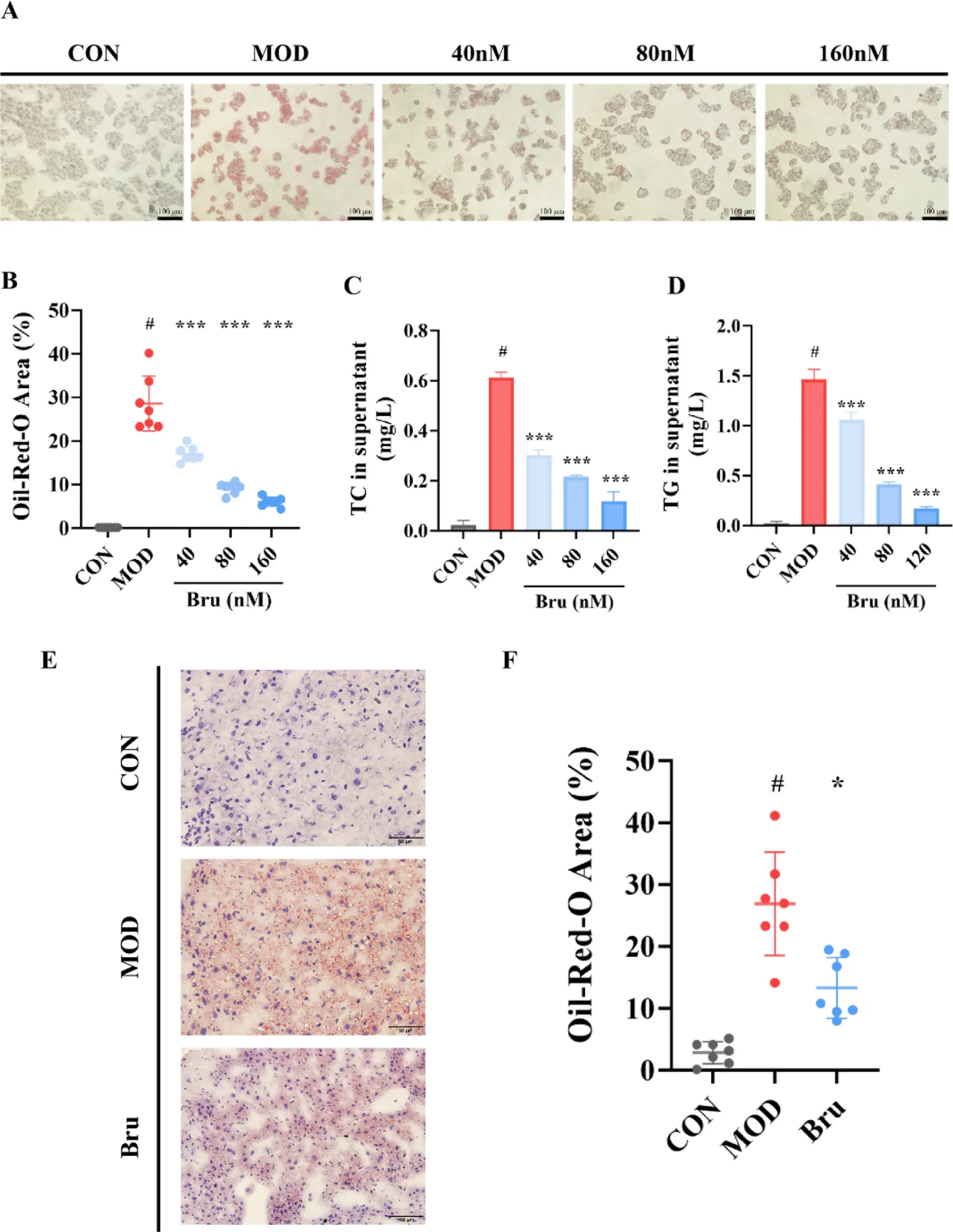
Brusatol suppresses lipid synthesis and accumulation in vitro and in vivo. **A** Representative Oil Red O-stained HepG2 cells induced with oleic acid (OA, 100 μM) and treated with Brusatol (40, 80, 160 nM) for 24 h. Lipid droplets are stained red; nuclei are counterstained with hematoxylin (blue). Scale bar: 50 μm. **B** Quantitative analysis of Oil Red O staining intensity in (A), normalized to the OA-induced model group. **C, D** Secreted levels of **C** total cholesterol (TC) and **D** triglycerides (TG) in the culture supernatant of HepG2 cells under the conditions described in (**A**). **E** Oil Red O staining of frozen liver sections from control (Con), IMQ-induced model (Mod), and Brusatol-treated (Bru) mice. Scale bar: 100 μm. **F** Quantification of lipid droplet area in liver sections based on (**E**). All data are expressed as mean ± SEM (n = 3 independent experiments for (**A**–**D**); n = 7 mice per group for (**F**)). ***p < 0.01 versus the OA-induced (**A**–**D**) or IMQ-induced Mod (**F**) group; #*p* < 0.01 versus the untreated control group

The original article [1] has been corrected.
