# Supplementary figures and images for: Brusatol ameliorates psoriatic dyslipidemia by targeting IL-1β to restore AMPK-mediated lipid homeostasis
Source: Chin Med. 2026 Jan 8;21:18. doi: 10.1186/s13020-025-01287-8 (PMC12781270; doi:10.1186/s13020-025-01287-8)

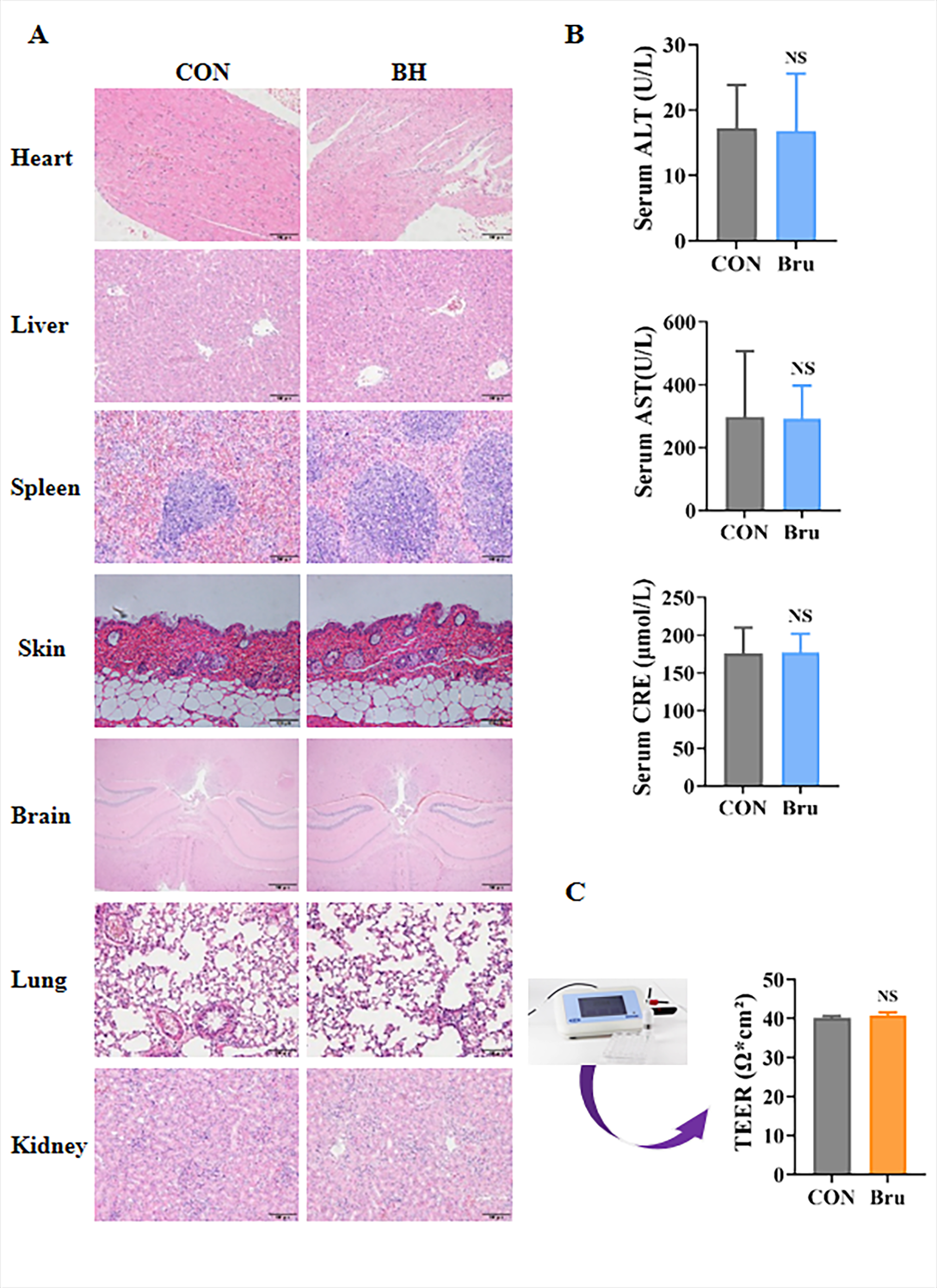

Supplement: Supplementary file 1 — Supplementary material 1. [file 13020_2025_1287_MOESM1_ESM.tif]
