# Supplementary material for: Brusatol ameliorates psoriatic dyslipidemia by targeting IL-1β to restore AMPK-mediated lipid homeostasis
Source: Chin Med. 2026 Jan 8;21:18. doi: 10.1186/s13020-025-01287-8 (PMC12781270; doi:10.1186/s13020-025-01287-8)
Supplement: Supplementary file 2 — Supplementary material 2. [file 13020_2025_1287_MOESM2_ESM.docx]

**Supplementary material**

**Brusatol ameliorates psoriatic dyslipidemia by targeting IL-1β to restore AMPK-mediated** **lipid** **homeostasis**

Yuankuan Jiang ^1,&^, Shumeng Zhang ^1,&^, Hewen Guan ^1,&^, Kejia lv ^1^, Jinchao Yu ^1^, Siyi Li ^1^, Renchuan Jia ^1^, Xiujie Zhang ^2^, Shurong Ma ^1^, Jialin Qu ^1,*^, Jingrong Lin ^2,^[[1]](#footnote-1)^*^

^1^ *Laboratory of Integrative Medicine, The First Affiliated Hospital of Dalian Medical University; No. 222, Zhongshan Road, Dalian 116011, China*

^2^ *Department of Dermatology, The First Affiliated Hospital of Dalian Medical University; No. 222, Zhongshan Road, Dalian 116011, China*

**Legends for Figures and Tables**

**Supplementary Fig.S1.** Predicted binding mode of brusatol within the IL-1β binding pocket obtained by molecular docking.

**Supplementary Table.S1.** The primer sequence of target gene.

**Supplementary Table S2**. Calculated Binding Free Energies (ΔG) of Brusatol to Various Human IL-1β Structural Conformations.

**Supplementary Table S3.** Comparative Analysis of Brusatol Binding to IL-1β and Its Upstream/Downstream Signaling Proteins


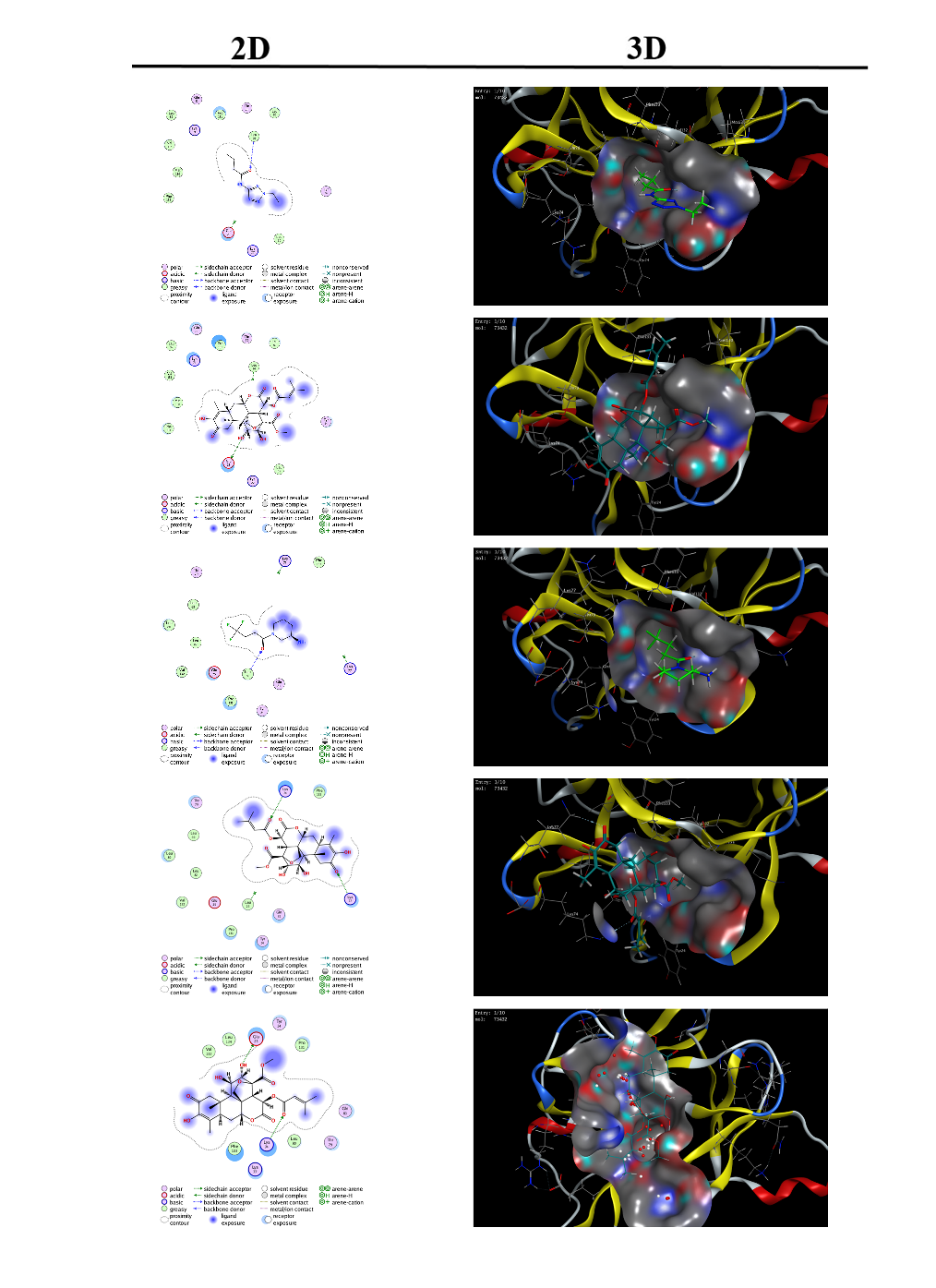


**Fig.S1.** Predicted binding mode of brusatol within the IL-1β binding pocket obtained by molecular docking.

**Table.S1.** The primer sequence of target gene.

Primer Sequence (5’ to 3’)

CPT1－F CACAACAACGGCAGAGCAGAG

CPT1－R ACACCACATAGAGGCAGAAGAGG

PPARα－F CACTTGCTCACTACTGTCCTTGG

PPARα－R TGCTGGTATCGGCTCAATAATTCC

SREBP-1c－F GGATCGCAGTCTGAGGAGGAG

SREBP-1c－R CCAGGAGCCGACAGGAAGG

ACC1－F CCTGCCACCTTATCACTATG

ACC1－R ACTGCCTGCCTGTCTCCATC

FASN－F TCCTGAAGCCGAACACCTCTG

FASN－R GCGACAATATCCACTCCCTGAATC

β-actin－F ACTGCCGCATCCTCTTCCTC

β-actin－R AACCGCTCGTTGCCAATAGTG

**Supplementary Table S2**. Calculated Binding Free Energies (ΔG) of Brusatol to Various Human IL-1β Structural Conformations.

| **PDB ID** | **Resolution (Å)** | **Ligand in Co-crystal** | **Binding Energy**  **of Brusatol (kcal/mol)** | **Binding Energy of Native Ligand (kcal/mol)** | **RMSD (Å)** |
| --- | --- | --- | --- | --- | --- |
| 5R8Q | 1.23 | 1-methyl-N-{[(2S)-oxolan-2-yl] methyl}-1H-pyrazole-3-carboxamide | -7.01 | -6.95 | 1.96 |
| 5R8E | 1.35 | ~{N}-(2-ethyl-1,2,3,4-tetrazol-5-yl) butanamide | -5.73 | -6.20 | 1.62 |
| 5R8M | 1.39 | 1-[(3~{R})-3-azanylpiperidin-1-yl]-4,4,4-tris(fluoranyl)butan-1-one | -6.24 | -6.22 | 1.30 |
| 8RYS | 1.16 | None | -6.13 | N/A | 1.51 |

**Supplementary Table S3.** Comparative Analysis of Brusatol Binding to IL-1β and Its Upstream/Downstream Signaling Proteins

| **Protein** | **Binding Energy (kcal/mol)** | **Note / Comparison to IL-1β** |
| --- | --- | --- |
| IL-1β | -7.01 | < -5 kcal/mol |
| NF-κb | -4.34 | > -5 kcal/mol |
| Casepase-1 | -4.83 | > -5 kcal/mol |
| TNF-α | -4.78 | > -5 kcal/mol |
| Gasdermin D | -4.66 | > -5 kcal/mol |

1. *^*^Correspondence should be addressed to Jingrong Lin (*[*Amy4963@163.com*](mailto:Amy4963@163.com)*)*

   *Co-correspondence author should be addressed to Jialin Qu (*[*jialin_qu@126.com*](mailto:jialin_qu@126.com)*)*

   ^&^ *These authors have contributed equally to this work*  [↑](#footnote-ref-1)
